# Identification of robust RT-qPCR reference genes for studying changes in gene expression in response to hypoxia in breast cancer cell lines

**DOI:** 10.1186/s12864-025-11216-6

**Published:** 2025-01-21

**Authors:** Jodie R. Malcolm, Katherine S. Bridge, Andrew N. Holding, William J. Brackenbury

**Affiliations:** 1 Department of Biology, University of York, York, YO10 5DD UK; 2 York Biomedical Research Institute, University of York, York, YO10 5DD UK; 3 Centre for Blood Research, University of York, York, YO10 5DD UK

## Abstract

Hypoxia is common in breast tumours and is linked to therapy resistance and advanced disease. To understand hypoxia-driven breast cancer progression, RT-qPCR is a widely used technique to quantify transcriptional changes that occur during malignant transformation. Reference genes (RGs) are endogenous RT-qPCR controls used to normalise mRNA levels, allowing accurate assessment of transcriptional changes. However, hypoxia reprograms transcription and post-transcriptional processing of RNA such that favoured RGs including *GAPDH* or *PGK1* are unsuitable for this purpose. To address the need for robust RGs to study hypoxic breast cancer cell lines, we identified 10 RG candidates by analysing public RNA-seq data of MCF-7 and T-47D (Luminal A), and, MDA-MB-231 and MDA-MB-468 (triple negative breast cancer (TNBC)) cells cultured in normoxia or hypoxia. We used RT-qPCR to determine RG candidate levels in normoxic breast cancer cells, removing *TBP* and *EPAS1* from downstream analysis due to insufficient transcript abundance. Assessing primer efficiency further removed *ACTB*,* CCSER2* and *GUSB* from consideration. Following culture in normoxia, acute, or chronic hypoxia, we ascertained robust non-variable RGs using RefFinder. Here we present *RPLP1* and *RPL27* as optimal RGs for our panel of two Luminal A and two TNBC cell lines cultured in normoxia or hypoxia. Our result enables accurate evaluation of gene expression in selected hypoxic breast cancer cell lines and provides an essential resource for assessing the impact of hypoxia on breast cancer progression.

**Supplementary Information:**

The online version contains supplementary material available at 10.1186/s12864-025-11216-6.

## Introduction

Breast cancer is the most common malignancy diagnosed worldwide. Due to advancements in therapeutic strategies and early detection, overall survival has greatly improved over the last few decades [1]. However, approximately one third of diagnoses will result in death as a consequence of chemotherapeutic resistance, metastasis, or delayed presentation of treatment toxicities [2–4]. Therefore, identifying novel molecular targets for therapeutic intervention is imperative. Current hormonal therapies targeting oestrogen receptor (ERα) activity have been effectively used to treat ERα positive (ERα+) Luminal A and Luminal B breast cancers, which account for ~ 70% of diagnoses [5]. However, breast cancers that co-express ERα and human epidermal growth factor receptor 2 (HER2), HER2 alone, or triple negative breast cancers (TNBC), which do not express any hormone or growth factor receptors, are more aggressive and tougher to treat. Moreover, acquired or *de novo* resistance to ERα-targeting drugs is an additional barrier that further diminishes survival for women with ERα+ disease [6].

Solid tumours, including those of the breast, have regions of limited O_2_ availability (hypoxia) due to increased O_2_ consumption in rapidly dividing cancer cells, and inadequate perfusion and diffusion of O_2_ as cells outgrow local blood vessel supply [7, 8]. Hypoxia-inducible factors (HIF)-1α and HIF-2α accumulate in hypoxic cells and are key transcriptional regulators of the hypoxic response. HIF-α subunits are constitutively expressed, even when O_2_ is abundant. However, under physiological levels of O_2_, HIF-α proteins are rapidly degraded by the proteasome via a tightly regulated process involving prolyl hydroxylase domain (PHD) enzymes and von Hippel-Lindau protein (pVHL) [9]. PHD enzymes use O_2_ as a catalytic substrate to hydroxylate HIF-α proteins, and are inhibited under hypoxic conditions; this in turn inhibits proteasomal degradation, and promotes accumulation of HIF-α subunits [10]. Stabilised HIF-α subunits translocate into the nucleus whereby they form heterodimers with HIF-1β and bind to hypoxia response elements (HREs) present within promoters of target genes, initiating transcription. In solid tumours, hypoxia and HIFs are recognised as important contributors to cancer progression and metastasis [11]. Hypoxia has been shown to remodel the chromatin landscape of breast cancer cells to promote epithelial-to-mesenchymal transition (EMT) in a HIF-1α-dependent manner [12]. Additionally, hypoxia has been linked to chemotherapy and radiotherapy resistance, and poor disease outcomes [13–15].

To assess complex physiological changes occurring during hypoxia-mediated breast cancer progression and therapy resistance, reverse transcription - quantitative real-time polymerase chain reaction (RT-qPCR) is gold standard for accurately quantifying gene transcription and capturing dynamic changes in gene expression that may be serving as molecular drivers of advanced disease [16]. A fundamental component of RT-qPCR is inclusion of reference genes (RGs) which act as internal controls for endogenous normalisation of measured target gene expression. RGs are selected on the basis of constitutive expression, and relative abundance not being altered by experimental conditions [17]. The substantial adjustment to the epigenome and transcriptome of cells that occurs under hypoxic conditions renders traditional RGs such as glycolytic enzymes *GAPDH* or *PGK1* redundant; despite this, comprehensive, systematic determination of RGs for hypoxia studies has yet to be performed [18–21].

We sought to fill this important knowledge gap by identifying RGs suitable for interrogating effects of hypoxia in breast cancer, using four widely cited breast cancer cell lines representing both ERα+ Luminal A (MCF-7 and T-47D) and TNBC (MDA-MB-231 and MDA-MB-468) subtypes. We identified 10 RG candidates following analysis of a publicly available RNA-seq dataset [22, 23]. We then established a comprehensive investigation of candidates to determine RGs with the least variability in expression after being cultured in normoxia (20% O_2_), acute hypoxia (1% O_2_, 8 h) or chronic hypoxia (1% O_2_, 48 h). RG candidates not abundantly expressed or associated with poor primer efficiencies were filtered out during the selection process. RGs were chosen by employing the web-based RG tool RefFinder [24, 25]. Our findings identify *RPLP1*, or *RPLP1* in combination with *RPL27*, as optimal RGs for analysis of hypoxia-mediated gene transcription in MCF-7, T-47D, MDA-MB-231 and MDA-MB-468 breast cancer cell lines. Our identification of *RPLP1* and *RPL27* as robust RGs in this panel of hypoxic Luminal A and TNBC cell lines provides a valuable resource for future studies investigating important transcriptional changes occurring during breast cancer progression.

## Materials and methods

### Cell culture

MCF-7, T-47D, MDA-MB-231 and MDA-MB-468 cell lines were used in this investigation. All breast cancer cell lines were maintained in DMEM (Gibco; S41966-029) supplemented with 5% foetal bovine serum (FBS; Gibco; 10270106) in a humidified Binder CO_2_ incubator at 37 °C and 5% CO_2_. Cells were regularly tested for *Mycoplasma* by immunofluorescent visualisation of *Mycoplasma* DNA with DAPI [26]. T-47D cells were provided by Dr. Andrew Holding (University of York), originally from ATCC, and MDA-MB-231 cells were a gift from Prof. Mustafa Djamgoz (Imperial College London). Both T-47D and MDA-MB-231 cell lines were authenticated by commercial STR profiling [27]. The MCF-7 and MDA-MB-468 cell lines were purchased from ATCC. For hypoxia culture, breast cancer cell lines were incubated in a humidified Baker Ruskinn InvivO_2_ oxygen workstation (37 °C, 1% O_2_, 5% CO_2_) for 8–48 h.

### Selection of RG candidates

High throughput RNA-seq datasets of 32 breast cancer cell lines cultured in 20% or 1% O_2_ for 24 h are available from the NCBI Gene Expression Omnibus (GEO; Series Accession: GSE111653) [22, 23]. Using the University of York’s Viking 2 cluster, we recovered paired-end fastq files for hypoxic and normoxic MCF-7, T-47D, MDA-MB-231 and MDA-MB-468 breast cancer cells with *fastq-dump* (Supplementary Table S1). Low-quality reads were trimmed with *trimmomatic* (ILLUMINACLIP: TruSeq3-PE.fa:2:30:20 LEADING:3 TRAILING:3 SLIDINGWINDOW:4:15 MINLEN:36) and fastQC reports were generated with *fastQC*. Reads were pseudo aligned to the GRCh38.p14 annotation (release 111) and quantified using *kallisto.* Hierarchical Data Format (h5) files containing quantified reads for each experiment were input into RStudio (version 4.3.3). Here, quantified reads were aggregated on the gene level using *sleuth_prep (gene_mode* = TRUE) for differential analysis.

To determine relative stability across a selection of common RGs, and generate a shortlist of RG candidates, normalised reads in transcript per million (TPM) at common RGs in hypoxia and normoxia were assessed independently for each of the four cell lines. A shortlist of RG candidates was selected based on (i) the appearance of the RG in literature searches and/or (ii) a calculated similarity (s) score of ≤ 0.30 between the 20% and 1% O_2_ conditions in at least two of the breast cancer cell lines. s was calculated by s = 1 - MIN(A, B) / MAX(A, B) (Microsoft Excel), where A is the read count value for a gene in 1% O_2_, B is the read count value for a gene in 20% O_2_, MIN refers to the smallest value between A and B and MAX determines the maximum value between A and B.

### RNA isolation and cDNA synthesis

Breast cancer cell lines were seeded to a density of 0.2 × 10^6^ per well in 6-well plates and were left for a minimum of 24 h to adhere to the surface of wells before starting experiments. Each experiment was carried out with three biological replicates, consisting of three technical replicates. The experiments were designed such that all samples from each normoxic or hypoxic timepoint were collected on the same day. RNA isolation, cDNA synthesis and RT-qPCR were performed in accordance with MIQE guidelines where appropriate [28]. At the experiment endpoint, cold QIAzol lysis reagent (QIAgen; 79306) was used to harvest RNA, as per manufacturer’s guidelines. Samples were rapidly collected in QIAzol, placed on ice and stored at -80 °C before RNA extraction. For phase separation, phenol/chloroform extraction with isopropanol precipitation was carried out as previously described [29]. To enhance nucleic acid extraction, GlycoBlue Coprecipitant (Invitrogen; AM9515) was included in the isolation protocol. Nucleic acid was re-suspended in 0.2 μm-filtered RNase-free water (Ambion; AM9937) and treated with DNase I (New England BioLabs; M0303S) to remove contaminating genomic DNA. RNA concentration and purity were measured using a NanoDrop™ One/OneC Microvolume UV-Vis Spectrophotometer (Thermo Fisher Scientific). RNA with an A260/280 of ≥ 2.0 was used. To ensure integrity, RNA was assessed by 1.5% agarose gel electrophoresis in denaturing conditions.

RNA was reverse transcribed using SuperScript IV cDNA Synthesis Kit as per manufacturer’s instructions (Invitrogen; 18091050). The amount of total RNA was 1 µg. The reaction volume was 20 µl and consisted of 1 µl 0.1 M DTT, 4 µl SSIV buffer, 1 µl RNAseOUT, 1 µl SSIV Enzyme, 1 µg of RNA in 11 µl of dH_2_O, 1 µl of random hexamer and 1 µl of 10 mM dNTP. Reactions were carried out on a Bioer LifePro thermocycler, comprising an initial step at 65 °C for 05:00 (mm: ss), followed by 23 °C for 10:00 (mm: ss), 55 °C for 10:00 (mm: ss), 80 °C for 10:00 (mm: ss) and then 4 °C for 10:00 (mm: ss). cDNA samples were diluted to 5 ng / µl in 0.2 μm-filtered RNase-free water (Ambion; AM9937). A standard curve was prepared from pooled RNA from each biological replicate, and diluted to 20 ng / µl, 4 ng / µl, 0.8 ng / µl, 0.16 ng / µl and 0.032 ng / µl. Samples were stored at -30 °C until further downstream analysis.

### RT-qPCR

RT-qPCR was performed using the QuantStudio™ 7 qPCR system (Thermo Fisher) in MicroAmp optical 384-well reaction plates (Applied Biosystems; 4309849) sealed with Expell™ optical sealing membranes (CAPP; 510400 C). Technical reactions were performed in duplicate using 2X SYBR Green SuperMix (Applied Biosystems; 4385612). Each reaction mixture had a final working volume of 12 µl, containing 6 µl SuperMix, 1 µl 10 µM primer stock (Table 1) and 4 µl of 5 ng / µl cDNA. Primer sequences for *ACTB* [30], *RPL27* [31], *CCSER2* [32], *GUSB* [33], *TFRC* [34, 35] and *CA9* [36] have been described before. For *OAZ1*, *TBP*, *RPL30*, *RPLP1*, *PGK1* and *EPAS1*, NCBI Primer BLAST was used to generate primer pair sequences that span the exon-exon junction with an amplicon size of between 70 and 200 bp and an optimal melting temperature of 60 ± 3 °C. All primer sequences were run through NCBI Primer BLAST to ensure no unintended gene targets could be amplified, but predicted transcript variants of the same gene were allowed. Primers were purchased from Integrated DNA Technologies.

For the standard curve, 80 ng, 16 ng, 3.2 ng, 0.64 ng and 0.128 ng of pooled cDNA were used. A no-template reaction was included as a negative control. RT-qPCR cycling parameters comprised an initial denaturation step at 95 °C for 01:35 (mm: ss), followed by 40 cycles of 00:03 (mm: ss) at 95 °C and 00:30 (mm: ss) at 60 °C. Melt curve analysis was carried out in the final cycle of the RT-qPCR by increasing the temperature from 60 °C to 95 °C at 0.1 °C per second.

**Table 1:** Primer sequences and protein features of reference genes and hypoxia responders

Uniprotkb	Gene	Protein	Forward Primer Sequence	Reverse Primer Sequence	Amplicon Length (bp)	Source	Biological Function
P54368	*OAZ1*	Ornithine decarboxylase antizyme 1	ATAAACCCAGCGCCACCATC	AGGGAGACCCTGGAACTCTCA	97	This study	Regulator of cell growth & proliferation
P60709	*ACTB*	β-Actin	CCTCGCCTTTGCCGATCC	GGATCTTCATGAGGTAGTCAGTC	626	^30^	Regulator of cell motility & structure
P20226	*TBP*	TATA-binding protein	GTGAGGTCGGGCAGGTTC	AAGAAACAGTGATGCTGGGTCA	108	This study	Essential regulator of gene transcription
P61353	*RPL27*	Ribosomal protein L27	ATCGCCAAGAGATCAAAGATAA	TCTGAAGACATCCTTATTGACG	123	^31^	Structural constituent of ribosome
P62888	*RPL30*	Ribosomal protein L30	ACTGCCCAGCTTTGAGGAAAT	GCCACTGTAGTGATGGACACC	77	This study	Structural constituent of ribosome
P05386	*RPLP1*	60 S acidic ribosomal protein P1	AGGAAGCTAAGGCTGCGTTG	GCATTGATCTTATCCTCCGTGACT	180	This study	Important in elongation during translation
Q9H7U1	*CCSER2*	Coiled-Coil Serine Rich Protein 2	GACAGGAGCATTACCACCTCAG	CTTCTGAGCCTGGAAAAAGGGC	143	^32^	Predicted: microtubule binding & bundling
P08236	*GUSB*	β-Glucuronidase	CTGTACACGACACCCACCAC	ATTCGCCACGACTTTGTT	159	^33^	Degrades glycosaminoglycans in the lysosome
P02786	*TFRC*	Transferrin receptor 1	GGACGCGCTAGTGTTCTTCT	CATCTACTTGCCGAGCCAGG	126	^34^	Ion uptake via receptor-mediated endocytosis
P00558	*PGK1*	Phosphoglycerate kinase 1	GGAGCTCCTGGAAGGTAAAGTC	TCCTGGCACTGCATCTCTTG	185	This study	Glycolytic enzyme used in glucose metabolism
Q99814	*EPAS1*	Endothelial PAS domain-containing protein 1	CACCTCGGACCTTCACCACC	TCCTCTCCGAGCTACTCCTTTTC	160	This study	Regulator of oxygen-dependent gene transcription
Q16790	*CA9*	Carbonic anhydrase IX	GTGCCTATGAGCAGTTGCTGTC	AAGTAGCGGCTGAAGTCAGAGG	115	^36^	Maintaining intracellular and extracellular pH

### Determining RG stability in breast cancer cell lines in normoxia, or acute or chronic hypoxia

Following RT-qPCR, reaction summaries were exported from ThermoFisher Design and Analysis Data Gallery and analysed in Microsoft Excel. A standard curve was used to calculate primer efficiency (PE) using the equation PE%$\:\:=(1{0}^{(-1/m)}-1)*100$

where $\:m$ denotes the slope of the standard curve. Then, PE$\:\:=SUM(PE\%/100)+1$. Where PE was > 2.20 or < 1.80, RG candidates were excluded from further analysis. Efficiency-corrected Ct values (CtE) were calculated using the equation

CtE$$\:\:=\:SUM\left(Ct*\right(Log\left(PE\right)/Log\left(2\right)\left)\right)$$.

mRNA expression (mE) of normoxic RGs was determined by mE$\:\:=\:1{0}^{\left(\right(CtE\:-\:a)/m)}$ where $\:a$ refers to the Y intercept.

CtE values were supplied to the online tool RefFinder for determination of the most stable reference genes to be used in normoxic vs. hypoxic breast cancer cell lines (available at https://www.ciidirsinaloa.com.mx/RefFinder-master) [24]. The RefFinder program employs the computational RG analysis tools geNorm [37], Normfinder [38], BestKeeper [39] and the comparative ΔCt method [40] to rank candidate RGs based on the individual ranking from each of the RG analysis tools.

### Validation of RGs

The change in expression of the HIF-regulated, hypoxia-induced target gene *CA9* was assessed using the 2^−ΔΔCt^ method [41], with the geometric mean of the recommended RG combination for comparative analysis between MCF-7, T-47D, MDA-MB-231 and MDA-MB-468 breast cancer cell lines used for normalisation. One-way ANOVA and Dunnett’s multiple comparisons were performed to assess significant fold-change in *CA9* expression following normalisation with the geometric mean of the recommended pair of RGs. Statistical analysis was performed using GraphPad Prism. Significance was reported where *p* < 0.05.

## Results

### Analysis of public RNA-seq data identifies ten RG candidates

The aim of our study was to identify optimal RGs for investigations of normoxic vs. hypoxic ERα+ Luminal A (MCF-7 and T-47D) and TNBC (MDA-MB-231 and MDA-MB-468) cell lines. We selected cell lines based on widespread use in breast cancer research: MCF-7, T-47D and MDA-MB-231 represent more than two-thirds of cell lines used within such studies [42].

To address the need for robust RGs, we first used a publicly available RNA-seq dataset that investigated genome-wide transcriptional changes taking place in 32 breast cancer cell lines as a consequence of O_2_ deprivation [22, 23]. From the 30,187 genes quantified in selected ERα+ (MCF-7 and T-47D) and TNBC (MDA-MB-231 and MDA-MB-468) cell lines, we were able to evaluate overall Euclidean distance between individual datasets (Supplementary Figure S1) and responsiveness of hypoxia-regulated genes to ensure cell lines behaved as expected when cultured in the absence of O_2_. Analysis demonstrated increased expression of *CA9*,* PGK1* and *VEGFA* in all four cell lines in response to hypoxic culture, and in agreement with previous findings (Supplementary Figure S2) [20, 43–45]. We also looked at *ARNT* (HIF-1β), *ARNT2* (HIF-2β), *EPAS1*,* HIF1A* and *HIF3A* expression in each of the four cell lines in normoxia and hypoxia (Supplementary Figure S3). Interestingly, we found *EPAS1*, the gene encoding HIF-2α, appeared to be relatively stable in expression in TNBC but not ERα+ cell lines (Supplementary Figure S3).

Next, we interrogated read count stability of common RGs when ERα+ and TNBC cells were cultured in hypoxia or normoxia, to identify RG candidates that may be stably expressed in each cell line, regardless of O_2_ availability (Supplementary Figures S4 and S5). From this, we generated a shortlist of 10 RG candidates (Table 2). We initially selected candidates based on common use as RGs in breast cancer cell lines (e.g. *CCSER2* in MCF-7, T-47D, MDA-MB-231 and MDA-MB-468 cell lines), or as stable RGs in other models of hypoxia (e.g. *RPLP1* in hypoxic pre-conditioned human neural stem cells) [46–48], and further stratified candidates based on a calculated similarity score (s) which was used to determine the similarity of read counts in genes from breast cancer cell lines cultured in 20% or 1% O_2_. Where $\:s$ = 0, read counts are the same between the two conditions. A minimum threshold was established, where s ≤ 0.30 in at least two of the cell lines, for an RG candidate to be carried forward.

ERα+ MCF-7 cells had the greatest variability in expression of the 10 RG candidates, compared to T-47D and the TNBC cell lines, with *CCSER2*,* EPAS1*,* OAZ1* and *TFRC* exceeding the maximum threshold for RG candidate selection, with s scores of 0.39, 0.33, 0.34 and 0.39, respectively (Table 2). Additionally, when s was calculated across the transcriptome of each breast cancer cell line, MCF-7 cells had the highest percentage of genes exceeding the maximum threshold set as a marker of stable gene expression (Supplementary Figure S6). *EPAS1* also responded positively to hypoxic culture in T-47D cells with an s score of 0.40, whereas no induction was observed in the TNBC cells. However, *EPAS1* was the only RG candidate that exceeded the maximum threshold in T-47Ds. Furthermore, for MDA-MB-231 and MDA-MB-468 cell lines, only *TFRC* or *TBP* displayed altered expression following O_2_ deprivation, with s scores of 0.32 and 0.34, respectively. The remaining RG candidates *ACTB*,* GUSB*,* RPL27*,* RPL30* and *RPLP1* were stably expressed across the two conditions, in all cell lines (Table 2; Supplementary Figure S4).

**Table 2:** Similarity (s) score between hypoxic and normoxic RNA-sequencing reads (TPM) of RG candidates

RG Candidate	Cell Line	Normoxia (TPM)	Hypoxia (TPM)	s Score
*ACTB*	MCF-7	2414.55	1860.14	0.23
	T-47D	4207.65	3874.77	0.08
	MDA-MB-231	4351.13	4864.34	0.11
	MDA-MB-468	4303.52	6144.26	0.30
*CCSER2*	MCF-7	12.72	20.99	0.39
	T-47D	20.55	19.81	0.04
	MDA-MB-231	31.95	27.11	0.15
	MDA-MB-468	28.21	28.32	0.00
*EPAS1*	MCF-7	0.60	0.88	0.33
	T-47D	35.53	21.35	0.40
	MDA-MB-231	28.94	26.96	0.07
	MDA-MB-468	58.87	57.13	0.03
*GUSB*	MCF-7	40.36	45.53	0.11
	T-47D	84.66	68.09	0.20
	MDA-MB-231	48.25	44.31	0.08
	MDA-MB-468	40.41	51.16	0.21
*OAZ1*	MCF-7	1167.01	775.30	0.34
	T-47D	769.99	687.38	0.11
	MDA-MB-231	1216.07	1275.52	0.05
	MDA-MB-468	881.12	908.12	0.03
*RPL27*	MCF-7	1901.03	2499.22	0.24
	T-47D	1726.21	1964.37	0.12
	MDA-MB-231	1883.42	1773.80	0.06
	MDA-MB-468	944.38	1049.75	0.10
*RPL30*	MCF-7	5177.74	7321.43	0.29
	T-47D	2884.35	3093.66	0.07
	MDA-MB-231	1937.35	1852.97	0.04
	MDA-MB-468	1258.62	1252.28	0.01
*RPLP1*	MCF-7	2982.47	3504.12	0.15
	T-47D	1367.30	1748.70	0.22
	MDA-MB-231	1478.45	1492.10	0.01
	MDA-MB-468	955.97	1024.99	0.03
*TBP*	MCF-7	16.28	18.87	0.14
	T-47D	11.24	9.14	0.19
	MDA-MB-231	14.54	16.06	0.09
	MDA-MB-468	16.38	10.76	0.34
*TFRC*	MCF-7	163.80	99.57	0.39
	T-47D	578.39	795.26	0.27
	MDA-MB-231	120.05	177.34	0.32
	MDA-MB-468	158.89	154.56	0.03

### Eight candidate RGs are highly expressed in normoxic breast cancer cells

**Fig. 1:**
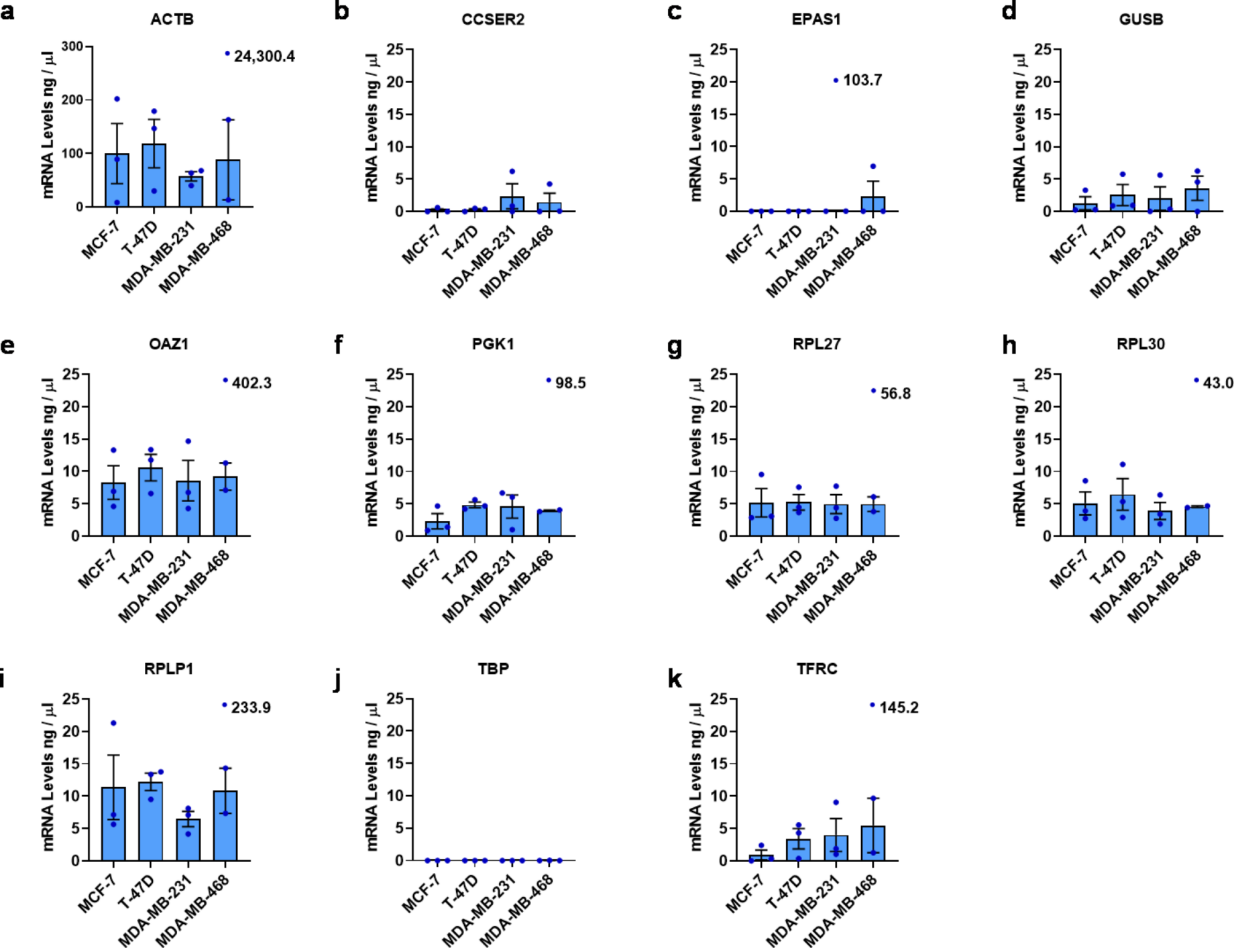
Expression of RG candidates in MCF-7, T-47D, MDA-MB-231 and MDA-MB-468 breast cancer cell lines cultured in 20% O_2_. Selected RG candidates (**A**) *ACTB*, (**B**) *CCSER2*, (**C**) *EPAS1*, (**D**) *GUSB*, (**E**) *OAZ1*, (**F**) *PGK1*, (**G**) *RPL27*, (**H**) *RPL30*, (**I**) *RPLP1* (**J**) *TBP* and (**K**) *TFRC* were evaluated for mRNA expression in breast cancer cell lines cultured in normal conditions for 72 h post seeding. Error bars are ± SEM. *n* = 3. Where there is an outlier, data point is displayed above the relevant box plot with mRNA expression value included

To demonstrate suitability of RG candidates, we used RT-qPCR to confirm RG expression in TNBC and ERα+ breast cancer cell lines cultured in normal O_2_ conditions. *ACTB* was expressed most highly among the breast cancer cell lines, but also showed greatest variation between biological replicates ranging from 8 ng / µl to 202 ng / µl in MCF-7 cells, and 30 ng / µl to 179 ng / µl in T-47D cells (Fig. 1A). *EPAS1* was only amplified in one biological replicate in MDA-MB-231 and MDA-MB-468 cells, with mRNA levels of 103 ng / µl and 6 ng / µl, respectively (Fig. 1C). Additionally, *TBP* did not have detectable levels of transcript in any cell lines (Fig. 1J). These results are supported by the RNA-seq analysis (Supplementary Figures S3 and S4), where TPM for *EPAS1* and *TBP* were among the lowest in expression in the breast cancer cell lines compared to other RG candidates. We therefore removed *TBP* and *EPAS1* from further investigation. The next lowest expressed RG was *CCSER2*, expressed at 0.21, 0.31, 1.15 and 2.36 ng / µl in MCF-7, T-47D, MDA-MB-468 and MDA-MB-231 cells, respectively (Fig. 1B). The remaining RG candidates (*GUSB*, *OAZ1*, *RPL27*, *RPL30*, *RPLP1* and *TFRC*) and *PGK1* were more highly expressed in all cell lines (Fig. 1D-I, K).

### Robust RGs identified by evaluating ct values in normoxic vs. hypoxic breast cancer cells

**Fig. 2:**
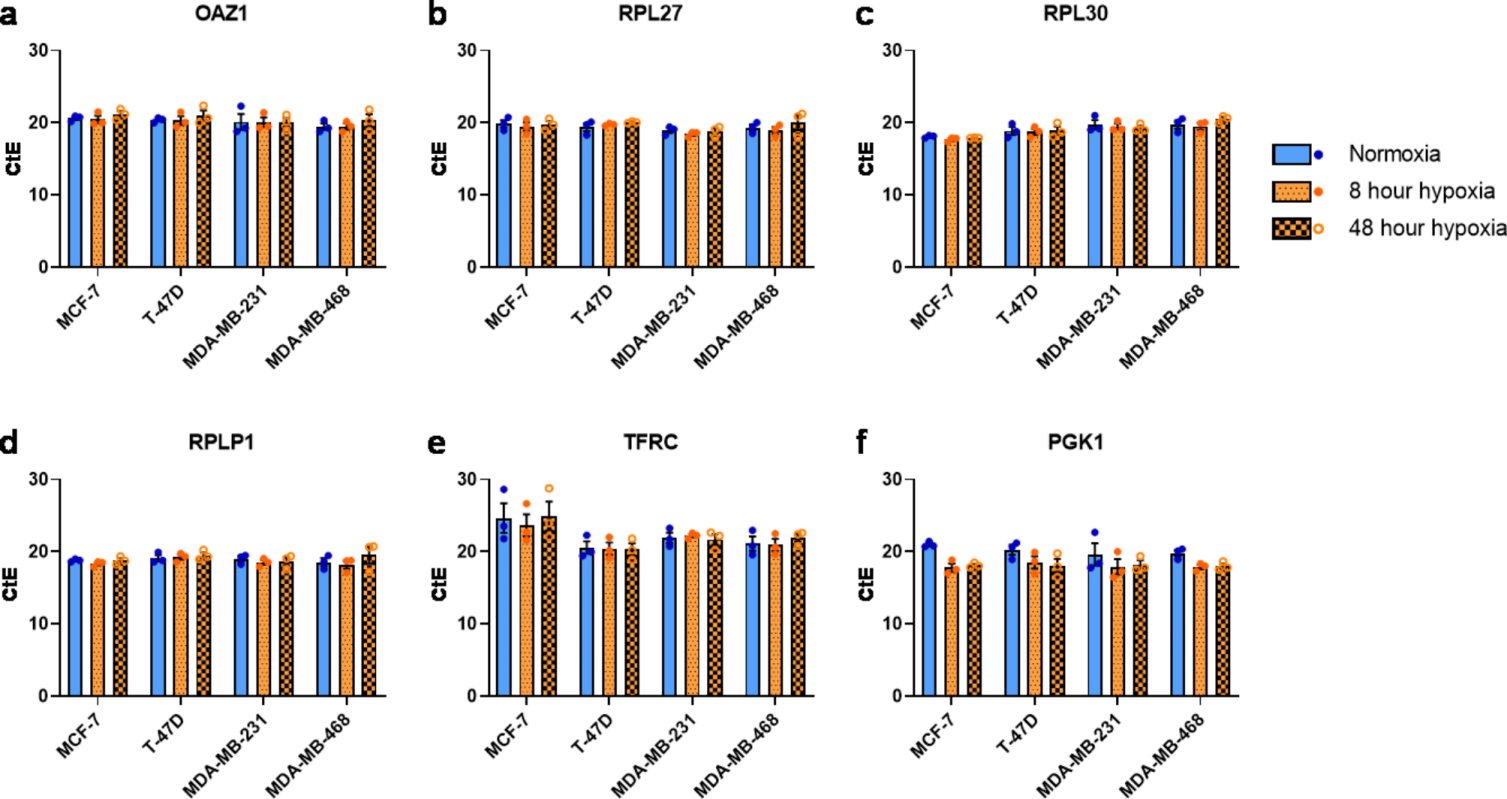
RG stability in breast cancer cell lines cultured in normoxia for 72 h post-seeding, or normoxia and then hypoxia for 8–48 h (total experimental time 72 h post-seeding). RT-qPCR was used to determine the variance in gene expression of selected RG candidates: (**A**) *OAZ1*, (**B**) *RPL27*, (**C**) *RPL30*, (**D**) *RPLP1*, (**E**) *TFRC* and (**F**) *PGK1* following culture of MDA-MB-231, MDA-MB-468, MCF-7 or T-47D breast cancer cell lines in normoxia (blue bars, closed blue points - left) or hypoxia for 8 h (orange bars, closed orange points - middle) or 48 h (orange checkered bars, open orange points - right). Error bars are ± SEM

Next, expression stability of RG candidates was investigated following culture in normoxia or hypoxia for 8–48 h (Fig. 2A; Supplementary Table S2). We also tested PEs from standard curves included in the RT-qPCR experiments (Supplementary Table S3; Supplementary Figures S7-S10). *ACTB*,* CCSER2* and *GUSB* displayed poor PE (Supplementary Table S3; *ACTB* mean 1.70, range 1.48–1.96; *CCSER2* mean 2.43, range 2.05–3.26; *GUSB* mean 2.22, range 2.03–2.50). These RG candidates were therefore removed from downstream analysis. *OAZ1*,* RPL27*,* RPL30* and *RPLP1* were expressed at comparatively similar levels across all cell lines, and in each condition (Fig. 2A-D). *TFRC* showed inter-cell line stability when cultured in normoxia, or acute or chronic hypoxia. However, intra-cell line CtE was more varied. In particular, *TFRC* had higher CtE values in MCF-7 cells, which suggests this gene is not as highly expressed in this cell line compared to the other cell lines (Fig. 2E). As predicted based on the literature, *PGK1* CtE values decreased in all cell lines following hypoxic culture for 8–48 h, implying that expression of *PGK1* increases in response to limited O_2_ supply (Fig. 2F). This result is in line with previous reports of hypoxic induction of *PGK1* [20, 47, 49, 50].

**Fig. 3:**
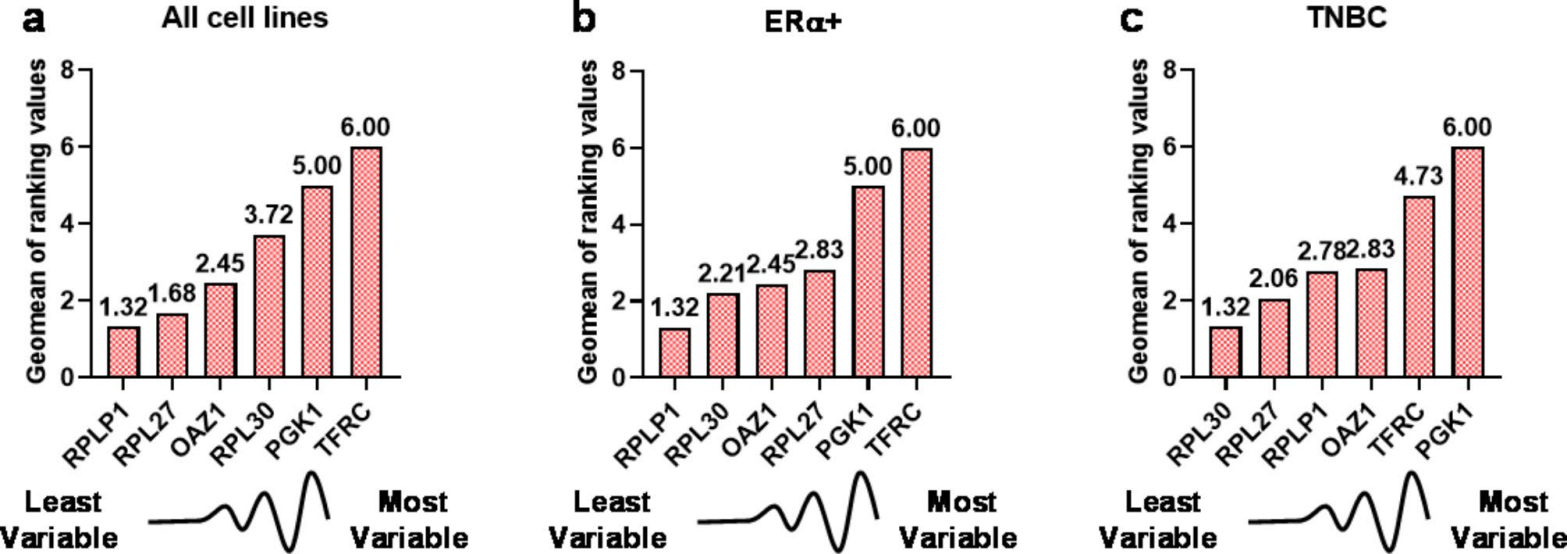
Geometric mean (Geomean) of ranking values for each RG candidate according to RefFinder. The final overall ranking of RG candidates was determined by RefFinder based on the geometric mean of the weights of each gene from GeNorm, NormFinder, BestKeeper and the comparative ΔCt method for (**A**) all breast cancer cell lines, (**B**) ERα+ breast cancer cell lines MCF-7 and T-47D and (**C**) TNBC cell lines MDA-MB-231 and MDA-MB-468

We then submitted CtE values (Supplementary Table S2) of the five remaining RG candidates, *OAZ1*,* RPL27*,* RPL30*,* RPLP1* and *TFRC*, as well as hypoxia-responder *PGK1*, to RefFinder, with intent to rank RG candidates in order of expression stability across all cell lines in normoxia or acute or chronic hypoxia. RefFinder first employs GeNorm, NormFinder, BestKeeper and the comparative ΔCt method to independently rank RGs. Next, RefFinder assigns a weight to an individual gene based on RG performance in the prerequisite programs, and calculates the geometric mean of candidate weights to provide a final ranking of the most stable RGs [24, 25]. In all iterations of RG stability analysis across all cell lines, *PGK1* and *TFRC* were ranked 5th and 6th, respectively (Supplementary Table S4). According to BestKeepeer and the comparative ΔCt method, *RPLP1* had the least variable inter- and intra-cell line expression in normoxic and hypoxic environments. *RPLP1* was also the highest ranked RG candidate by RefFinder (Fig. 3A). Conversely, NormFinder ranked *OAZ1* as the best RG candidate, and placed *RPL27* and *RPLP1* as the second and third best RG candidates (Supplementary Table S4). A benefit of GeNorm over the other programs is the additional assessment of the optimal number of RGs to use for accurate normalisation [37]. For the study of hypoxia-mediated alterations in gene expression between MCF-7, T-47D, MDA-MB-231 and MDA-MB-468 breast cancer cell lines, GeNorm recommended the combined use of *RPL27* and *RPLP1*.

We next identified optimal RGs to be used for RT-qPCR of hypoxic breast cancer cell lines following stratification into breast cancer subtypes. When CtE values from ERα+ breast cancer cell lines were supplied, *RPLP1* was again ranked top RG candidate with the least variability in expression, according to RefFinder, BestKeepeer and the comparative ΔCt method (Supplementary Table S5, Fig. 3B). Normfinder suggested *OAZ1* to be the optimal RG to use when investigating hypoxic induction of genes of interest in the ERα+ Luminal A breast cancer group. GeNorm recommended the combined use of *RPLP1* and *RPL30*, instead of *RPL27*, for all cell lines. *PGK1* and *TFRC* were ranked as the least stable RGs in all outputs, as before. For the TNBC group, *RPL30* was placed first by all programs (Supplementary Table S6, Fig. 3C), apart from GeNorm which recommended *RPL27* and *RPLP1*, the same as for all four breast cancer cell lines. Analysis of the individual cell lines cultured in normoxia, and acute or chronic hypoxia was also performed. Here, GeNorm identified *RPLP1* and *RPL27* as the least variable and most suitable RGs for MDA-MB-231 or MDA-MB-468 cell lines, but *RPL30* was ranked as the least variable single RG by RefFinder in both TNBC models. *RPLP1* and *RPL30* were the least variable and most suitable RGs for the T-47D or MCF-7 cell lines (Supplementary Tables S7-S10, Supplementary Figure S11).

*RPLP1 and RPL27 are suitable for normalising gene expression in a panel of normoxic vs. hypoxic ER*α*+ and TNBC cell lines*.

**Fig. 4:**
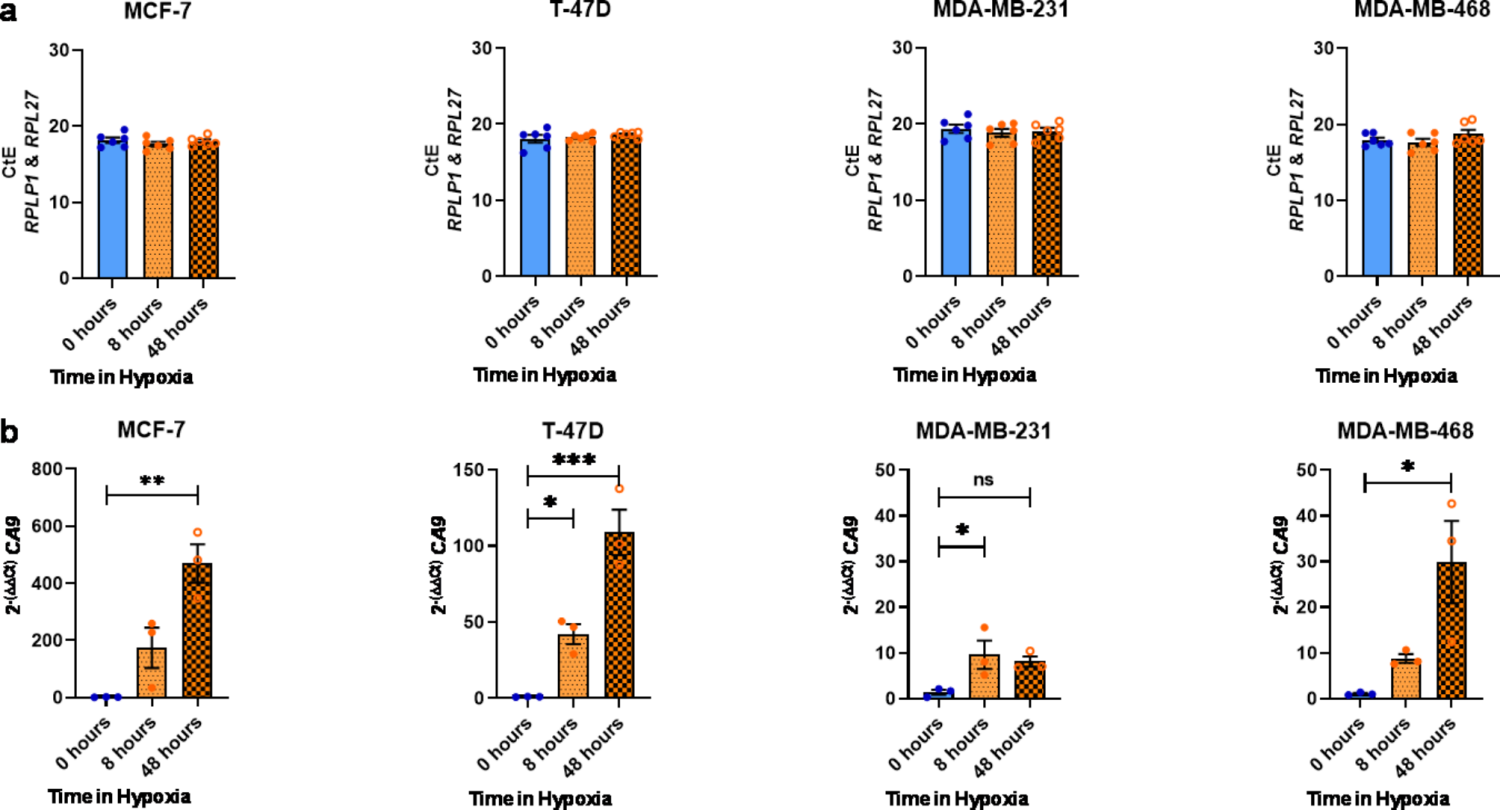
RG expression level stability and hypoxic induction of *CA9* in four breast cancer cell lines cultured in normoxia or hypoxia for 8–48 h. (**A**) *RPL27* (*n* = 3) and *RPLP1* (*n* = 3) expression was determined by RT-qPCR. Raw CtE values for triplicate biological replicates of the two RGs (*n* = 6) in MDA-MB-231, MDA-MB-468, T-47D and MCF-7 breast cancer cell lines are shown. Error bars are geometric mean ± geometric SD. (**B**) Expression of *CA9* was assessed in MCF-7, T-47D, MDA-MB-231 and MDA-MB-468 breast cancer cell lines following culture in normoxia (20% O_2_, “0 hours”) or hypoxia (1% O_2_) for 8–48 h. Changes in *CA9* expression were determined by the 2^−ΔΔCt^ method, using the geometric mean of RGs *RPLP1* and *RPL27* for normalisation (**A**). One-way ANOVA with Dunnett’s multiple comparisons was employed to investigate significance of fold change in gene expression relative to normoxic control. *p = < 0.05, **p = < 0.01, ***p = < 0.001. Error bars are ± SEM. *n* = 3

Following identification of optimal RGs, we aimed to evaluate combined use of *RPLP1* and *RPL27* for normalisation of gene transcription in a panel of normoxic and hypoxic breast cancer cell lines. We assessed upregulation of hypoxia-induced *CA9* in each breast cancer cell line cultured in normoxia or hypoxia for 8–48 h. The geometric mean of *RPLP1* and *RPL27* was used to normalise *CA9* CtE values, before fold change induction (2^−ΔΔCt^) of *CA9* was calculated [41]. Expression (CtE) of *RPLP1* and *RPL27* in MCF-7 (19.4 ± 0.4 SD), T-47D (19.7 ± 0.5 SD), MDA-MB-231 (18.8 ± 0.5 SD) and MDA-MB-468 (19.6 ± 0.9 SD) cells was consistent, regardless of environmental O_2_ (Fig. 4A). Conversely, all cell lines displayed significant induction of *CA9* following hypoxic culture (Fig. 4B). In MCF-7 cells, *CA9* was increased 470-fold after chronic exposure to a hypoxic environment. For T-47D cells, acute and chronic hypoxia induced a 42- and 109-fold increase in *CA9* expression, respectively. After 8 h of hypoxic culture, MDA-MB-231 cells showed a moderate but significant 9-fold induction, and for MDA-MB-468 cells a 17-fold increase in *CA9* expression occurred following 48 h of hypoxic culture. Importantly, *RPLP1* and *RPL27* were similarly expressed in each cell line, in each condition. Thus, combination of *RPLP1* and *RPL27* as RGs is suitable for normalising gene expression in this panel of normoxic and hypoxic breast cancer cell lines.

## Discussion

The use of RT-qPCR for investigating gene transcription has been customary practice in labs since quantitative PCR was first discussed by Higuchi et al. in 1993 [51]. While RT-qPCR is the gold standard for quantifying mRNA expression and understanding mechanisms involved in altered gene transcription, interpretation of gene expression is dependent on appropriate use of internal controls as a means of normalisation [52]. Common RGs previously deemed to have stable expression include *GAPDH*,* ACTB*,* PGK1* and *18 S rRNA*, which have subsequently been shown to have variation in abundance across different experimental conditions, emphasising the notion that there is no such thing as an RG that works for all investigations [53]. Indeed, in the context of cellular hypoxia, *ACTB* is affected by insufficient O_2_ supply, as are *GAPDH* and *PGK1* which are specifically regulated by the activity of HIF-1α [19–21, 50]. Thus, when looking to identify novel therapeutic targets to combat hypoxia-induced therapy resistance, suitable RGs need to be selected prior to RT-qPCR investigation of genes of interest, so that hypoxia-induced alterations in RG expression do not obscure novel and important biological findings.

To meet the demand for robust endogenous RGs for investigations of hypoxic ERα+ and TNBC cell lines, we carried out a comprehensive investigation combining bioinformatic analysis of publicly available RNA-seq datasets to select 10 RG candidates, RT-qPCR of those candidates to assess expression levels and variability, and utilisation of the online RG tool RefFinder to ensure the most suitable RGs were selected. The 10 RG candidates we identified included genes that are generally considered RGs (*ACTB*,* RPL30*,* RPLP1*,* GUSB*,* TBP* and *TFRC*), and novel RGs (*OAZ1*,* RPL27*,* CCSER2*, and *EPAS1*) [31, 46–48, 54, 55]. When CtEs of our candidates were supplied to RG selection tools, it is perhaps unsurprising that constituents of the ribosome (*RPLP1*,* RPL27* and *RPL30*) which are abundantly and consistently expressed in human tissues were selected as the optimal RGs with the least variability in expression in breast cancer cell lines cultured in normoxia, or acute or chronic hypoxia [56–58]. This result is supported by the observation that breast cancer cells can bypass hypoxia-mediated inhibition of protein synthesis through gene silencing of 4E-BP1, eEF2 kinase or tuberous sclerosis complex 2 (TSC2), maintaining a continuous requirement of translational machinery [59].

Throughout our study, we have chosen to include the process of RG candidate deselection, based on assessment of gene expression and primer efficiencies, as it is important to understand peripheral results which impact the quality of data interpretation. Thus, for full transparency of our RG selection process, we have shown negative filtration of poor candidates as well as positive selection of stable candidates. To ensure precision when normalising expression of genes of interest, we recommend including two RGs in RT-qPCR studies, as use of a single RG for normalising gene expression may result in erroneous interpretation, whereas inclusion of two RGs should ensure accurate normalisation of target gene abundance [28, 60].

With respect to selection of our 10 RG candidates, the RNA-seq dataset used to curate the shortlist was limited by a single replicate for each cell line in each condition being available for analysis [22, 23]. The original study is an impressive investigation into the molecular portrait of hypoxia spanning 32 breast cancer cell lines, and for the purpose of our study, provided a meaningful starting point for selecting and determining the approximate stability of RG candidates in our four chosen breast cancer cell lines.

A limitation of our study is that identification of ribosomal proteins as suitable RGs may only be applicable to those wishing to capture hypoxia-induced changes in gene expression in the breast cancer cell line panel investigated in this study (MCF-7, T-47D, MDA-MB-231, MDA-MB-468). How our results translate to other breast cancer cell lines, or indeed patient samples, requires further investigation. Cell lines representing the same disease model often display variation in response to environmental or experimental conditions and have unique gene expression signatures and molecular portraits [42]. This is exemplified in MCF-7 and T-47D cell lines, where 17β-oestradiol has been shown to confer disparate effects on gene expression between the two models of Luminal A breast cancer, despite both cell lines being driven by ERα activity [61]. For patient derived samples, the answer to identifying suitable RGs for RT-qPCR is more unclear, due to the complexity of individuality between patients, heterogeneity of cell types within the tumour microenvironment, and uneven distribution of hypoxia observed throughout tumours. Cancer grade at diagnosis, and samples coming from secondary metastatic sites, will also require further optimisation of RGs. Indeed, patterns of dysregulated ribosomal protein expression have been observed in human tissues, primary cell lines and tumours [62]. Thus, careful identification of suitable RGs for such studies needs to be implemented prior to carrying out experiments, and consideration given to including a greater number of RGs (3–5 for more complex tissue samples) would reduce variability and allow more accurate normalisation in such instances [37].

An alternative solution to normalising gene expression in more complex breast cancer specimens may be to incorporate spike-in controls of exogenous RG, of a known amount in the qPCR. In this case, RG transcripts can act as a stable reference, while simultaneously undergoing reverse transcription and amplification with the target transcripts [63]. Nonetheless, we have outlined a robust strategy for selection of suitable endogenous RGs that can be applied to a broad range of studies aiming to identify important transcriptional aberrations acting as drivers of breast cancer progression. Further, the method outlined in this study can serve as a best practice approach for selecting suitable RGs in experiments which may extend beyond the scope of hypoxia in breast cancer cells, such as those exploring hypoxia in the context of development, stroke, or heart failure.

In conclusion, we have carried out a comprehensive investigation to identify the most suitable RGs with the least variability in their expression, which can be used in RT-qPCR studies of MCF-7, T-47D, MDA-MB-231 and MDA-MB-468 breast cancer cell lines cultured in normoxia or hypoxia. We used robust computational RG selection programs following stringent criteria for identifying RG candidates and recommend the inclusion of *RPLP1* and *RPL27* in RT-qPCR studies as internal controls for accurate interpretation of gene expression results. Furthermore, this result provides the means to assess the impact of hypoxia within breast cancer development and progression when the chosen Luminal A and TNBC cell lines are utilised.

## Electronic supplementary material

Below is the link to the electronic supplementary material.

Supplementary Material 1

Supplementary Material 2

Supplementary Material 3

Supplementary Material 4

Supplementary Material 5

## Data Availability

All data supporting the findings of this study are available within the paper and its Supplementary Information. Raw qPCR data are provided in four supplementary xlsx files, one for each cell line. The datasets analysed during the current study are available in the NCBI GEO repository, GSE111653. Supporting code is available from https://zenodo.org/doi/10.5281/zenodo.13166160.

## Abbreviations

CtE: Efficiency-corrected Ct value
DMEM: Dulbecco’s modified eagle medium
EMT: Epithelial mesenchymal transition
ERα: Oestrogen receptor alpha
FBS: Foetal bovine serum
HER2: Human epidermal growth factor receptor 2
HIF: Hypoxia inducible factor
HRE: Hypoxia response element
PE: Primer efficiency
PHD: Prolyl hydroxylase domain
pVHL: von Hippel-Lindau protein
RG: Reference gene
RT-qPCR: Reverse transcription-quantitative PCR
TNBC: Triple negative breast cancer
TPM: Transcript per million
TSC2: Tuberous sclerosis complex 2
