# Supplementary material for: Identification of robust RT-qPCR reference genes for studying changes in gene expression in response to hypoxia in breast cancer cell lines
Source: BMC Genomics. 2025 Jan 21;26:59. doi: 10.1186/s12864-025-11216-6 (PMC11748566; doi:10.1186/s12864-025-11216-6)
Supplement: Supplementary file 1 — Supplementary Material 1 [file 12864_2025_11216_MOESM1_ESM.pdf]

Supplementary Table S1. SRA accession numbers of hypoxic (1% O_2, 24 hours) and normoxic breast cancer cell lines from BioProject PRJNA437670, series GSE111653

SRA Run Accession number	Breast Cancer Cell Line	Breast Cancer Subtype	Environmental Oxygen Status
SRR6822831	MCF-7	Luminal A	Hypoxic
SRR6822832			Normoxic
SRR6822837	MDA-MB-231-PSOC	Basal B	Hypoxic
SRR6822838			Normoxic
SRR6822841	MDA-MB-468	Basal A	Hypoxic
SRR6822842			Normoxic
SRR6822857	T-47D	Luminal A	Hypoxic
SRR6822858			Normoxic

Supplementary Table S2. Efficiency-corrected Ct values (*CtE*) of RGs following RT-qPCR of cells cultured in normoxic (**N**), acutely hypoxic (**A**; 8 hours) or chronically hypoxic (**C**; 48 hours) conditions. Biological replicates are listed as R1, R2 or R3 and correspond to the average Ct from three independent technical replicates per biological replicate.

	OAZ1	*RPL27*	*RPL30*	*RPLP1*	*TFRC*	*PGK1*	**Condition** and replicate
MDA-MB-231	18.80	19.43	19.21	19.48	20.76	18.42	**N** R1
	19.28	18.28	19.04	18.26	21.73	17.78	**N** R2
	22.31	19.11	21.02	19.28	23.26	22.70	**N** R3
	19.50	18.64	19.11	19.06	21.82	17.29	**A** R1
	19.33	18.67	19.07	18.54	22.28	16.47	**A** R2
	21.42	18.00	20.29	17.95	22.59	20.00	**A** R3
	18.96	19.43	18.93	19.38	20.24	17.54	**C** R1
	20.19	19.09	18.96	19.07	22.66	17.79	**C** R2
	21.11	17.97	19.92	17.60	22.22	19.38	**C** R3
MDA-MB-468	18.81	20.04	20.56	19.46	20.81	20.10	**N** R1
	19.28	19.47	18.66	18.63	19.59	18.93	**N** R2
	20.37	18.53	20.08	17.59	22.96	20.36	**N** R3
	18.75	19.13	20.03	18.78	20.65	18.04	**A** R1
	19.52	19.68	18.56	18.74	19.64	17.31	**A** R2
	20.19	18.08	19.93	17.08	22.52	18.35	**A** R3
	18.86	20.88	20.67	20.63	20.82	17.80	**C** R1
	21.84	21.25	20.92	20.73	22.60	18.66	**C** R2
	20.29	17.94	19.94	17.24	22.41	17.50	**C** R3
MCF-7	20.94	18.95	17.89	18.82	21.82	21.45	**N** R1
	20.19	20.75	18.16	19.00	23.54	20.72	**N** R2
	20.78	19.92	18.27	18.60	28.63	20.81	**N** R3
	21.51	18.47	17.92	18.60	21.61	18.87	**A** R1
	19.89	20.48	17.86	18.52	22.68	17.53	**A** R2
	20.00	19.54	17.34	17.90	26.67	17.00	**A** R3
	21.92	18.99	17.96	19.35	22.09	18.49	**C** R1
	20.42	20.58	17.88	18.73	23.96	18.04	**C** R2
	21.20	19.74	17.91	18.26	28.79	17.83	**C** R3
T-47D	20.70	18.31	18.79	18.61	19.45	20.65	**N** R1
	20.00	19.77	17.98	18.79	22.29	18.94	**N** R2
	20.46	20.11	19.89	19.94	19.98	21.22	**N** R3

	21.48	19.94	19.46	19.77	20.17	19.95	**A** R1
	20.07	19.58	18.01	18.57	22.02	16.95	**A** R2
	19.46	19.77	18.81	19.35	19.11	18.68	**A** R3
	22.39	20.22	20.00	20.26	20.24	19.75	**C** R1
	20.67	20.07	18.09	18.96	21.80	16.50	**C** R2
	20.02	19.97	18.69	19.30	18.92	17.97	**C** R3
Mean	20.30	19.41	19.05	18.86	22.04	18.77	
SE	0.17	0.14	0.17	0.14	0.37	0.25	
SD	1.00	0.87	1.00	0.83	2.23	1.49	
CV	0.05	0.04	0.05	0.04	0.10	0.08	

Supplementary Table S3: Efficiencies of RG primers

		ACTB	*CCSER2*	*GUSB*	*OAZ1*	*PGK1*	*RPL27*	*RPL30*	*RPLP1*	*TFRC*
MDA-MB-231		1.88	2.05	2.07	2.01	1.99	2.01	2.01	2.01	2.04
		1.64	2.19	2.23	1.99	1.94	1.97	2.05	1.96	2.18
		1.74	2.49	2.29	2.05	2.07	2.04	2.09	1.99	2.12
MDA-MB-468		1.96	2.08	2.03	2.02	1.98	2.02	2.06	1.98	2.01
		1.73	3.26	2.44	2.01	1.99	2.04	2.02	1.96	2.06
		1.51	2.39	2.50	1.99	1.96	1.99	2.04	1.90	2.21
MCF-7		1.95	2.16	2.05	2.03	2.02	2.01	2.03	1.98	2.07
		1.59	2.50	2.27	2.02	2.02	2.08	2.06	1.99	2.29
		1.48	2.99	2.33	2.03	1.97	2.01	2.04	1.93	2.63
T-47D		1.87	2.23	2.04	2.04	1.99	2.05	2.04	1.98	2.06
		1.49	2.52	2.24	2.00	1.95	2.09	2.00	1.96	2.42
		1.55	2.30	2.19	1.97	1.98	2.04	2.04	1.99	2.09
Mean		1.70	2.43	2.22	2.01	1.99	2.03	2.04	1.97	2.18
SE		0.03	0.06	0.02	0.00	0.01	0.01	0.00	0.00	0.03
SD		0.17	0.35	0.15	0.02	0.03	0.03	0.02	0.03	0.18
CV		0.10	0.14	0.07	0.01	0.02	0.02	0.01	0.01	0.08

Supplementary Table S4. Summary of individual rankings of RG stability according to RefFinder, comparative ΔCt method, BestKeeper, NormFinder and GeNorm. Ranking was determined by providing all CtE values for each RG assessed in **MCF-7, T-47D, MDA-MB-231** and **MDA-MB-468** breast cancer cell lines, in all conditions, to RefFinder.

	Ranking (1 = best - 6 = worst)					
Method	1	2	3	4	5	6
ΔCt	RPLP1	RPL27	OAZ1	RPL30	PGK1	TFRC
BestKeeper	RPLP1	RPL27	OAZ1	RPL30	PGK1	TFRC
Normfinder	OAZ1	RPL27	RPLP1	RPL30	PGK1	TFRC
GeNorm	RPL27 | RPLP1		RPL30	OAZ1	PGK1	TFRC
RefFinder recommended comprehensive ranking	RPLP1	RPL27	OAZ1	RPL30	PGK1	TFRC

Supplementary Table S5. Summary of individual rankings of RG stability according to RefFinder, comparative ΔCt method, BestKeeper, NormFinder and GeNorm. Ranking was determined by providing CtE values for each RG assessed in **MCF-7** and **T-47D ERα+** breast cancer cell lines, in all conditions, to RefFinder.

	Ranking (1 = best - 6 = worst)					
Method	1	2	3	4	5	6
ΔCt	RPLP1	RPL30	OAZ1	RPL27	PGK1	TFRC
BestKeeper	RPLP1	RPL27	RPL30	OAZ1	PGK1	TFRC
Normfinder	OAZ1	RPL27	RPLP1	RPL30	PGK1	TFRC
GeNorm	RPL30 | RPLP1		OAZ1	RPL27	PGK1	TFRC
RefFinder recommended comprehensive ranking	RPLP1	RPL30	OAZ1	RPL27	PGK1	TFRC

Supplementary Table S6. Summary of individual rankings of RG stability according to RefFinder, comparative ΔCt method, BestKeeper, NormFinder and GeNorm. Ranking was determined by providing all CtE values for each RG assessed in **MDA-MB-231** and **MDA-MB-468** TNBC cell lines, in all conditions, to RefFinder.

	Ranking (1 = best - 6 = worst)					
Method	1	2	3	4	5	6
ΔCt	RPL30	OAZ1	RPL27	RPLP1	TFRC	PGK1
BestKeeper	RPL30	RPL27	RPLP1	OAZ1	TFRC	PGK1
Normfinder	RPL30	OAZ1	RPL27	TFRC	RPLP1	PGK1
GeNorm	RPL27 | RPLP1		RPL30	OAZ1	TFRC	PGK1
RefFinder recommended comprehensive ranking	RPL30	RPL27	RPLP1	OAZ1	TFRC	PGK1

Supplementary Table S7. Summary of individual rankings of RG stability according to RefFinder, the comparative ΔCt method, BestKeeper, NormFinder and GeNorm. Ranking was determined by providing all CtE values for each RG assessed in **MCF-7** breast cancer cells, in all conditions, to RefFinder.

	Ranking (1 = best - 6 = worst)					
Method	1	2	3	4	5	6
ΔCt	*RPL30*	*RPLP1*	*OAZ1*	*RPL27*	*PGK1*	*TFRC*
BestKeeper	*RPL30*	*RPLP1*	*OAZ1*	*RPL27*	*PGK1*	*TFRC*
Normfinder	*RPL30*	*RPLP1*	*RPL27*	*OAZ1*	*PGK1*	*TFRC*
GeNorm	*RPL30* | *RPLP1*		*OAZ1*	*RPL27*	*PGK1*	*TFRC*
RefFinder recommended comprehensive ranking	*RPL30*	*RPLP1*	*OAZ1*	*RPL27*	*PGK1*	*TFRC*

Supplementary Table S8. Summary of individual rankings of RG stability according to RefFinder, the comparative ΔCt method, BestKeeper, NormFinder and GeNorm. Ranking was determined by providing all CtE values for each RG assessed in **T-47D** breast cancer cells, in all conditions, to RefFinder.

	Ranking (1 = best - 6 = worst)					
Method	1	2	3	4	5	6
ΔCt	*RPLP1*	*RPL30*	*RPL27*	*OAZ1*	*PGK1*	*TFRC*
BestKeeper	*RPL27*	*RPLP1*	*RPL30*	*OAZ1*	*TFRC*	*PGK1*
Normfinder	*RPLP1*	*RPL30*	*OAZ1*	*RPL27*	*PGK1*	*TFRC*
GeNorm	*RPL30* | *RPLP1*		*RPL27*	*OAZ1*	*PGK1*	*TFRC*
RefFinder recommended comprehensive ranking	*RPLP1*	*RPL30*	*RPL27*	*OAZ1*	*PGK1*	*TFRC*

Supplementary Table S9. Summary of individual rankings of RG stability according to RefFinder, the comparative ΔCt method, BestKeeper, NormFinder and GeNorm. Ranking was determined by providing all CtE values for each RG assessed in **MDA-MB-231** breast cancer cells, in all conditions, to RefFinder.

	Ranking (1 = best - 6 = worst)					
Method	1	2	3	4	5	6
ΔCt	*RPL30*	*OAZ1*	*TFRC*	*RPL27*	*RPLP1*	*PGK1*
BestKeeper	*RPL27*	*RPLP1*	*RPL30*	*TFRC*	*OAZ1*	*PGK1*
Normfinder	*RPL30*	*OAZ1*	*TFRC*	*RPL27*	*RPLP1*	*PGK1*
GeNorm	*RPL27 | RPLP1*		*RPL30*	*TFRC*	*OAZ1*	*PGK1*
RefFinder recommended comprehensive ranking	*RPL30*	*RPL27*	*RPLP1*	*OAZ1*	*TFRC*	*PGK1*

Supplementary Table S10. Summary of individual rankings of RG stability according to RefFinder, the comparative ΔCt method, BestKeeper, NormFinder and GeNorm. Ranking was determined by providing all CtE values for each RG assessed in **MDA-MB-468** breast cancer cells, in all conditions, to RefFinder.

	Ranking (1 = best - 6 = worst)					
Method	1	2	3	4	5	6
ΔCt	*RPL30*	*RPL27*	*OAZ1*	*RPLP1*	*PGK1*	*TFRC*
BestKeeper	*RPL30*	*OAZ1*	*PGK1*	*RPL27*	*RPLP1*	*TFRC*
Normfinder	*RPL30*	*OAZ1*	*RPL27*	*PGK1*	*RPLP1*	*TFRC*
GeNorm	*RPL27* | *RPLP1*		*RPL30*	*OAZ1*	*PGK1*	*TFRC*
RefFinder recommended comprehensive ranking	*RPL30*	*RPL27*	*OAZ1*	*RPLP1*	*PGK1*	*TFRC*

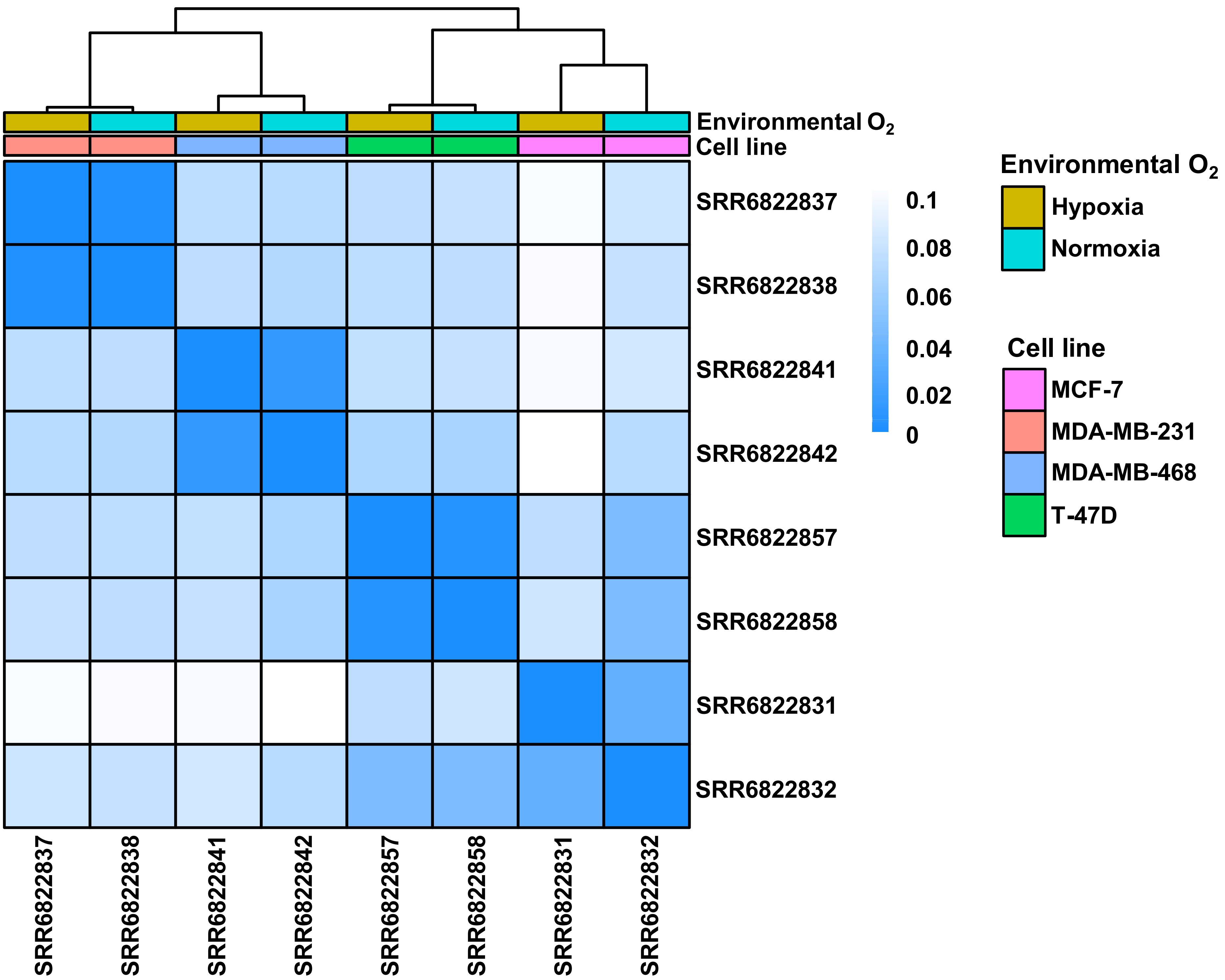

Supplementary Figure S1. Euclidean distance matrix (EDM) of squared distances between GSE111653 ERα+ and TNBC, hypoxic and normoxic breast cancer transcriptome datasets.

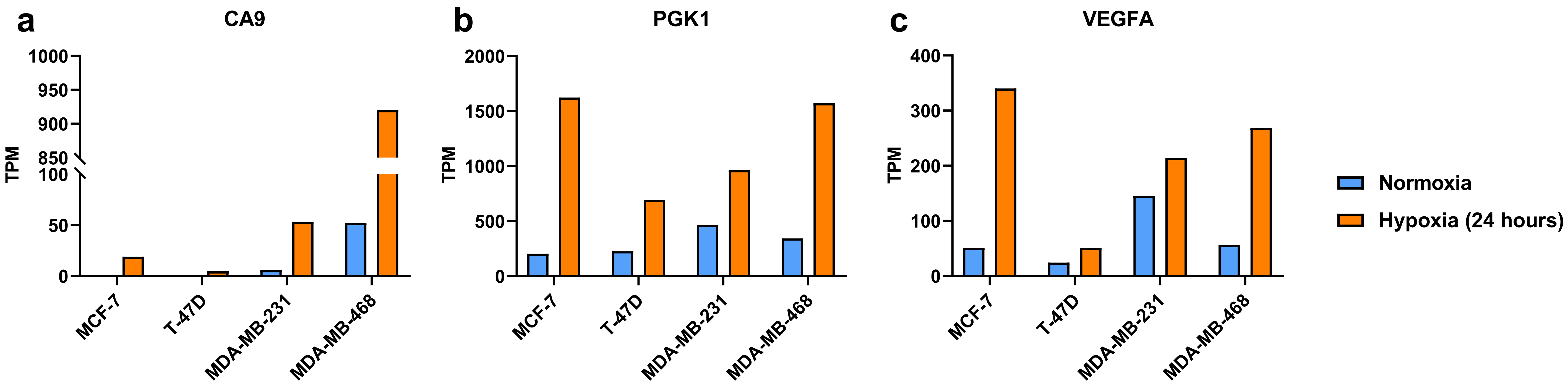

Supplementary Figure S2. High-throughput transcriptomic analysis of hypoxia-responsive genes (A) *CA9* and (B) *PGK1* and (C) *VEGFA* in breast cancer cell lines cultured in 20% O_2, (normoxia) or 1% O_2 (hypoxia) for 24 hours. The normalised read counts in transcripts per million (TPM) for each gene in MDA-MB-231, MDA-MB-468, MCF-7 and T-47D breast cancer cell lines cultured in normoxia (blue) or hypoxia (orange) are shown. Only one replicate was available for initial analysis.

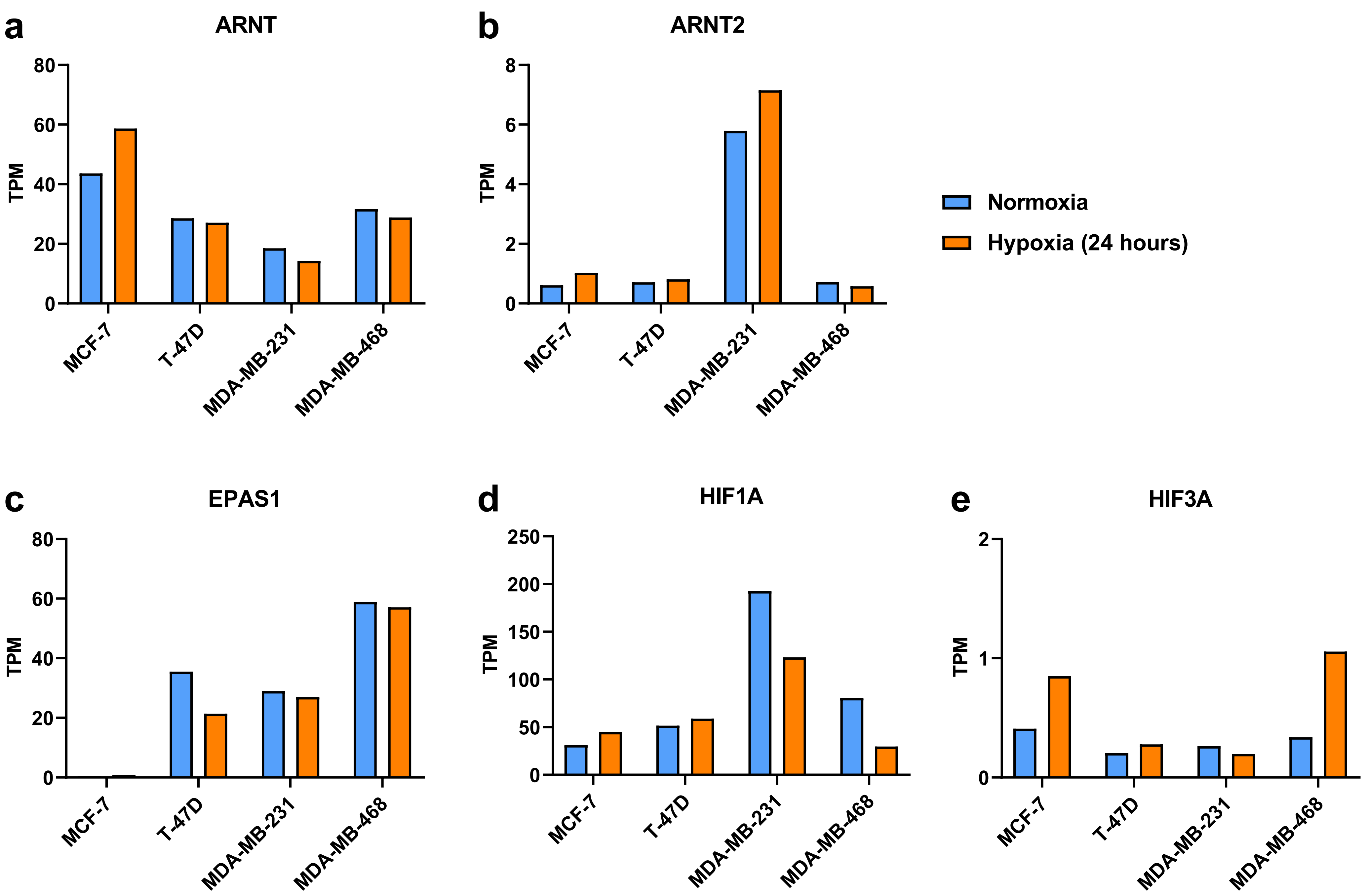

Supplementary Figure S3. High-throughput transcriptomic analysis of hypoxia-inducible factor (HIF) genes (A) *ARNT,* (B) *ARNT2,* (C) *EPAS1,* (D) *HIF1A* and (E) *HIF3A* in breast cancer cell lines cultured in 20% O_2, (normoxia) or 1% O_2 (hypoxia) for 24 hours. The normalised read counts in transcripts per million (TPM) for each gene in MDA-MB-231, MDA-MB-468, MCF-7 and T-47D breast cancer cell lines cultured in normoxia (blue) or hypoxia (orange) are shown. Only one replicate was available for initial analysis.

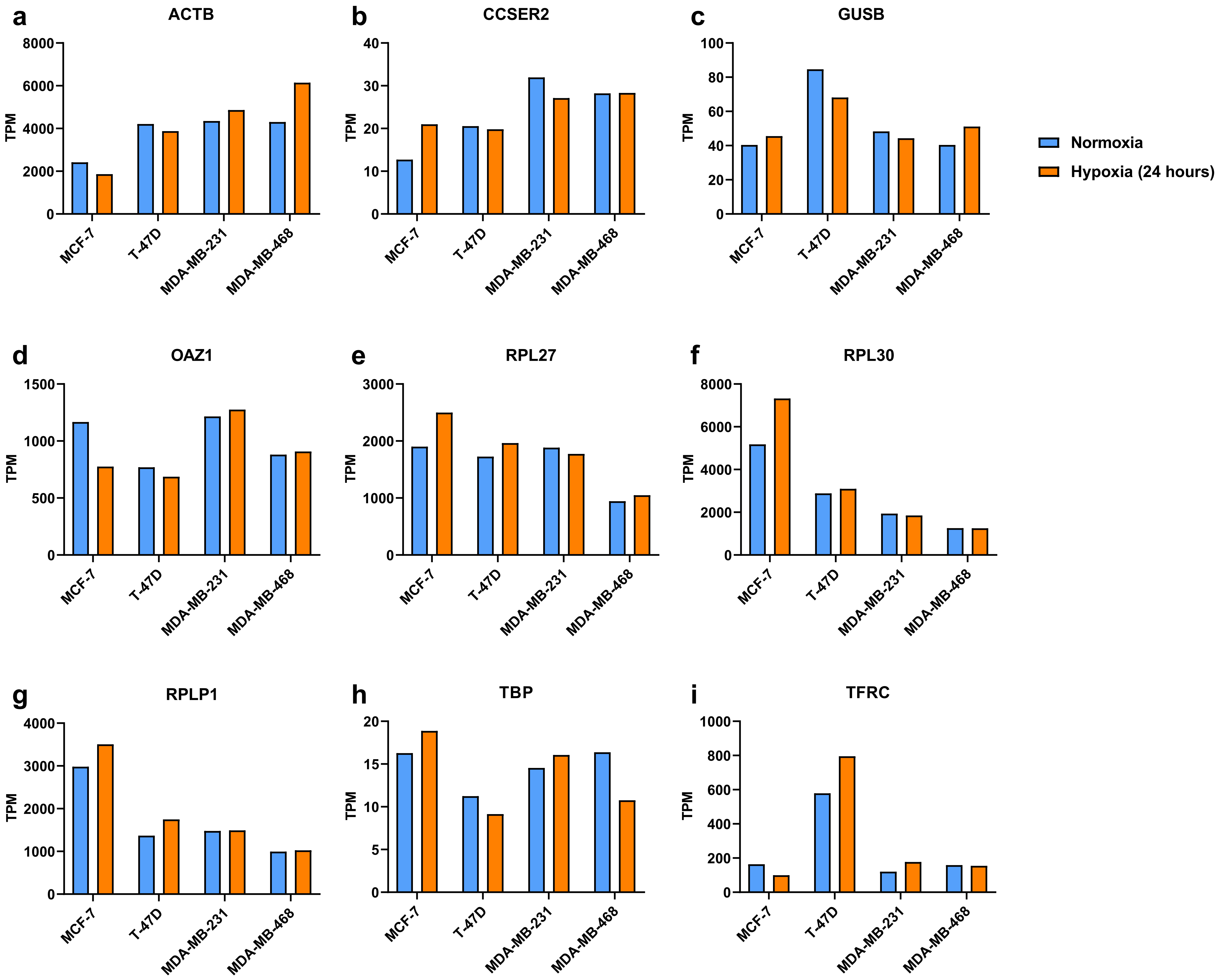

Supplementary Figure S4. High-throughput RNA-seq analysis of RGs in breast cancer cell lines cultured in 20% O_2, (normoxia) or 1% O_2 (hypoxia) for 24 hours. A shortlist of RGs to be assessed for their suitability for RT-qPCR normalisation included (A) *ACTB*, (B) *CCSER2*, (C) *GUSB*, (D) *OAZ1*, (E) *RPL27*, (F) *RPL30*, (G) *RPLP1* (H) *TBP* and (I) *TFRC*. The normalised read counts in transcripts per million (TPM) for each gene in MDA-MB-231, MDA-MB-468, MCF-7 and T-47D breast cancer cell lines cultured in normoxia (blue), or hypoxia (red) are shown. Only one biological replicate was available for analysis.

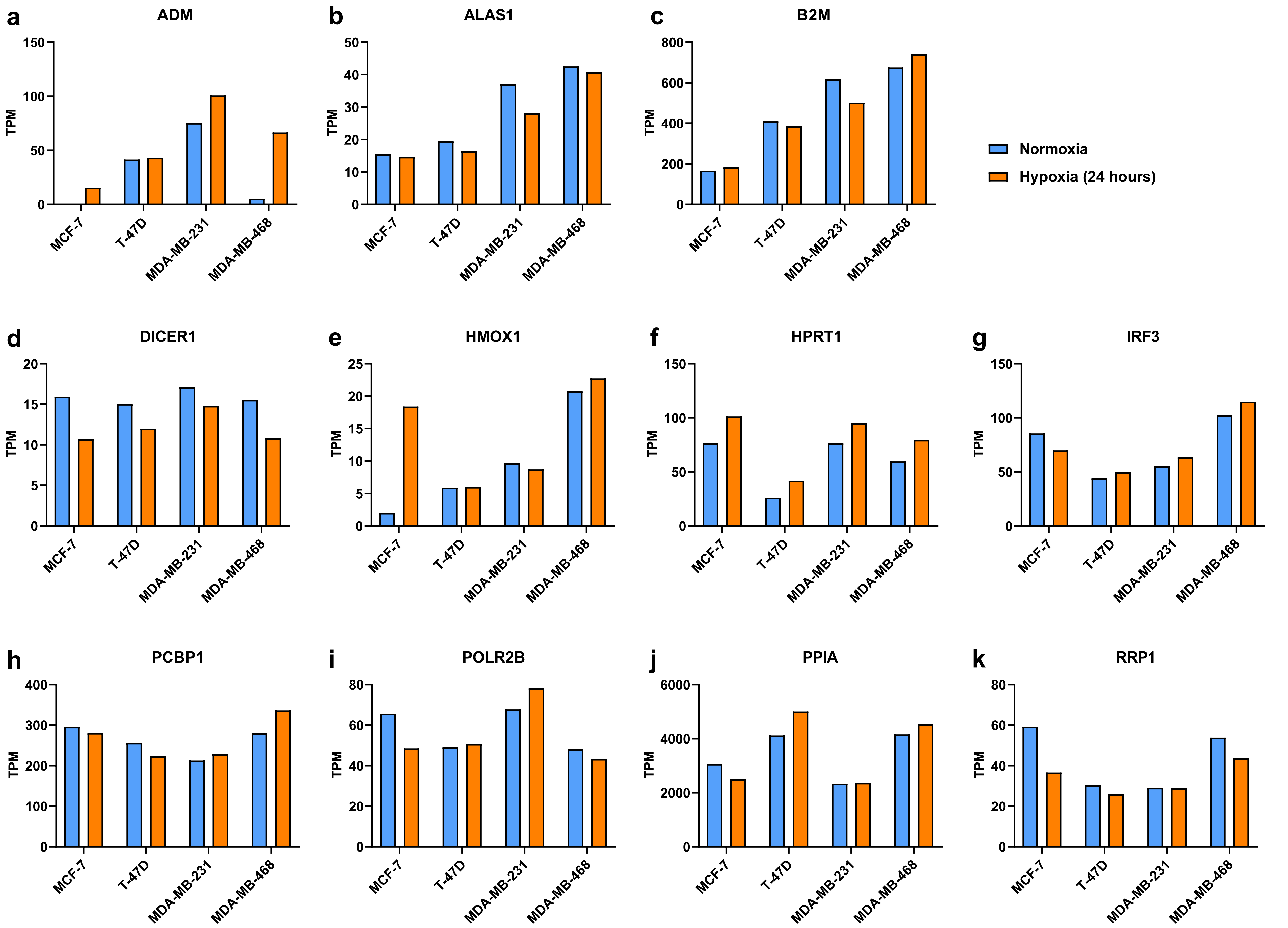

Supplementary Figure S5. High-throughput transcriptomic analysis of commonly used reference genes (A) *ADM,* (B) *ALAS1,* (C) *B2M,* (D) *DICER1,* (E) *HMOX1,* (F) *HPRT1,* (G) *IRF3,* (H) *PCBP1,* (I) *POLR2B,* (J) *PPIA* and (K) *RRP1* in breast cancer cell lines cultured in 20% O_2, (normoxia) or 1% O_2 (hypoxia) for 24 hours. The normalised read counts in transcripts per million (TPM) for each gene in MDA-MB-231, MDA-MB-468, MCF-7 and T-47D breast cancer cell lines cultured in normoxia (blue) or hypoxia (orange) are shown. Only one replicate was available for initial analysis.

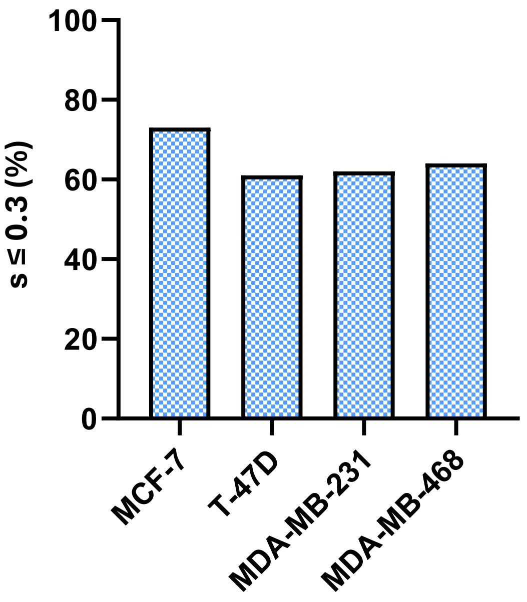

Supplementary Figure S6. *s* score analysis across all genes in breast cancer cell lines. For 30,187 genes, the percentage of genes that have an *s* score of equal to or greater than (≥) 0.3 has been calculated in each breast cancer cell line.

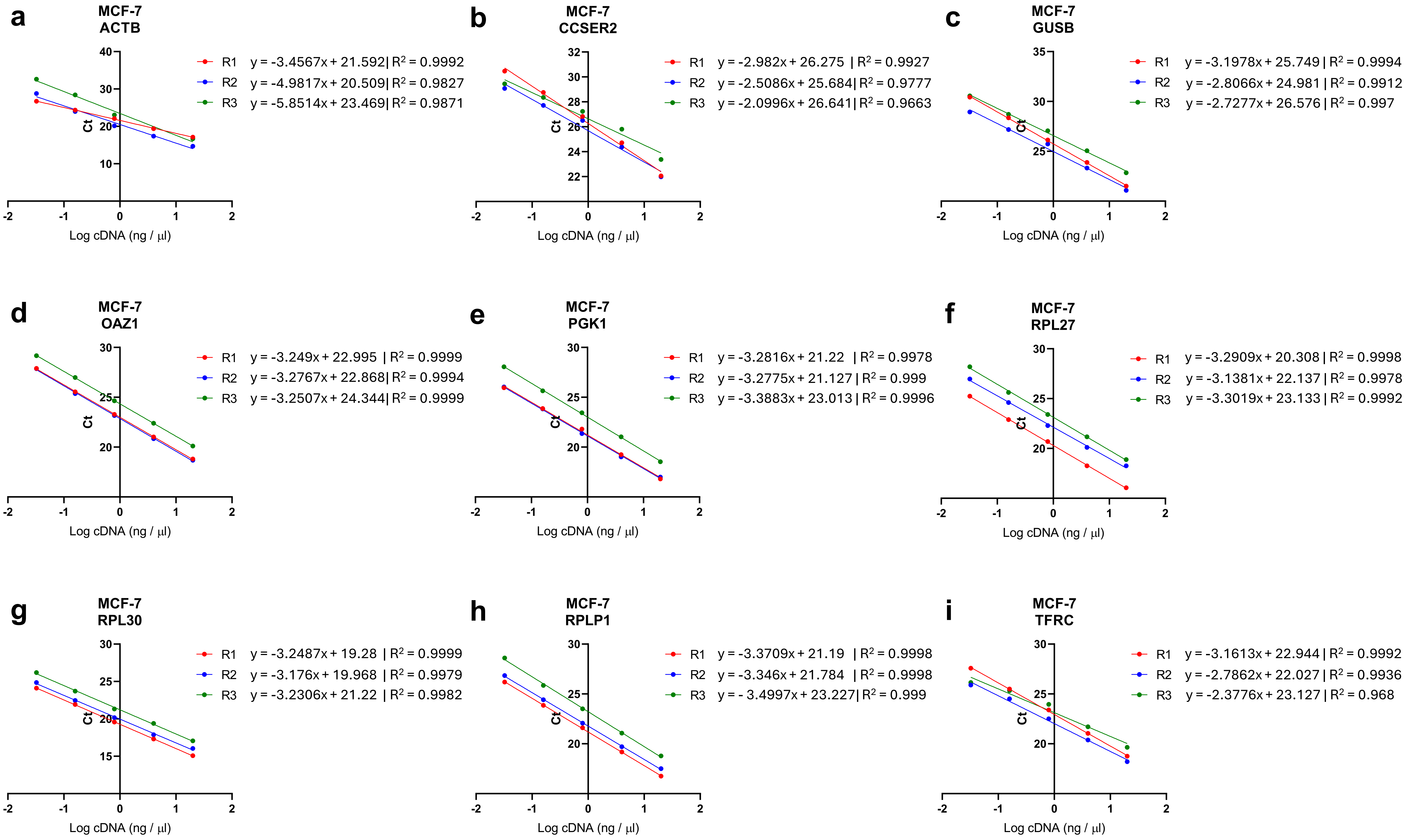

Supplementary Figure S7. Standard curves with line equations and R^2 value for (A) *ACTB,* (B) *CCSER2,* (C) *GUSB,* (D) *OAZ1,* (E) *PGK1,* (F) *RPL27,* (G) *RPL30,* (H) *RPLP1* and (I) *TFRC* in MCF-7 breast cancer cells. R1, R2 and R3 refers to the biological replicate.

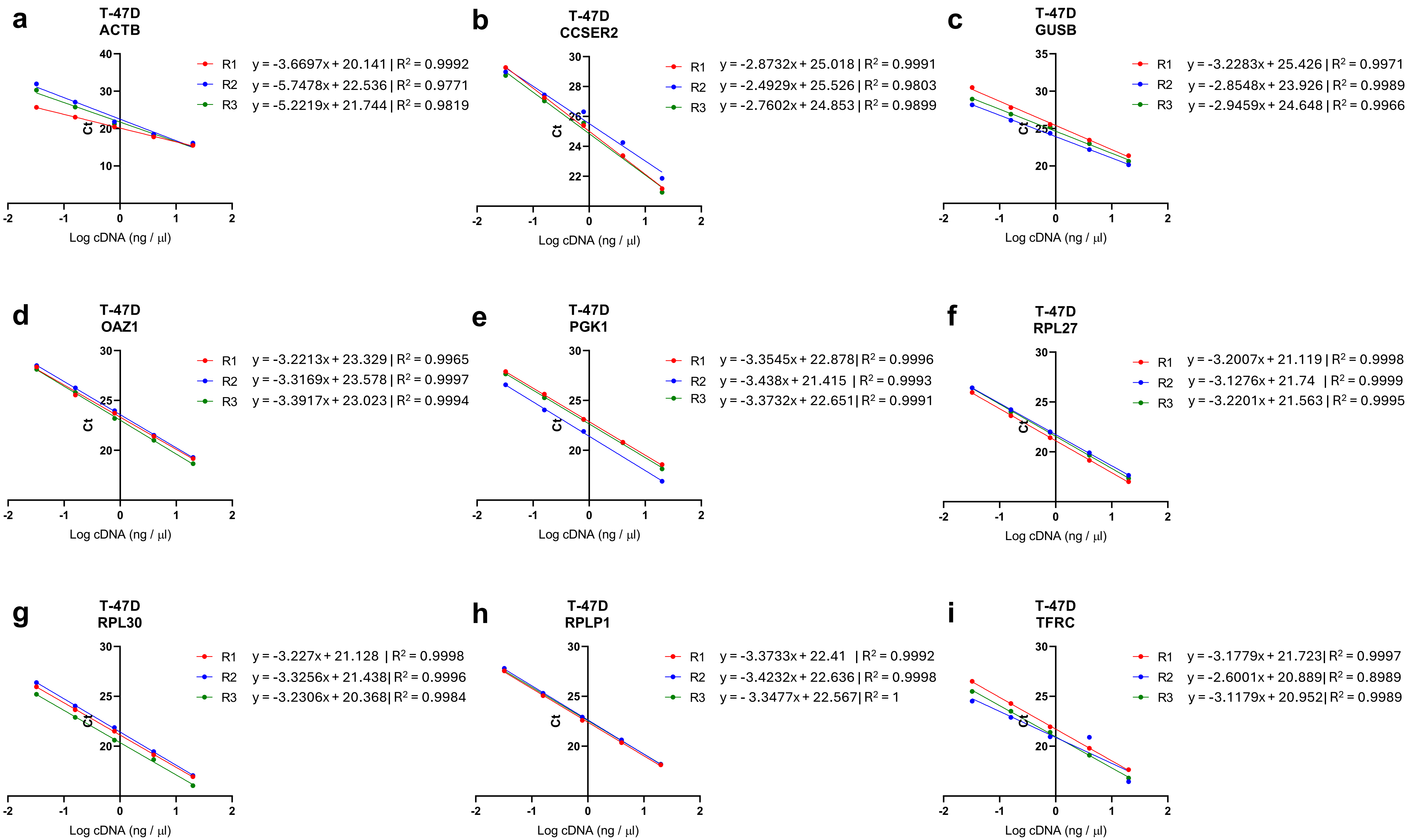

Supplementary Figure S8. Standard curves with line equations and R^2 value for (A) *ACTB,* (B) *CCSER2,* (C) *GUSB,* (D) *OAZ1,* (E) *PGK1,* (F) *RPL27,* (G) *RPL30,* (H) *RPLP1* and (I) *TFRC* in T-47D breast cancer cells. R1, R2 and R3 refers to the biological replicate.

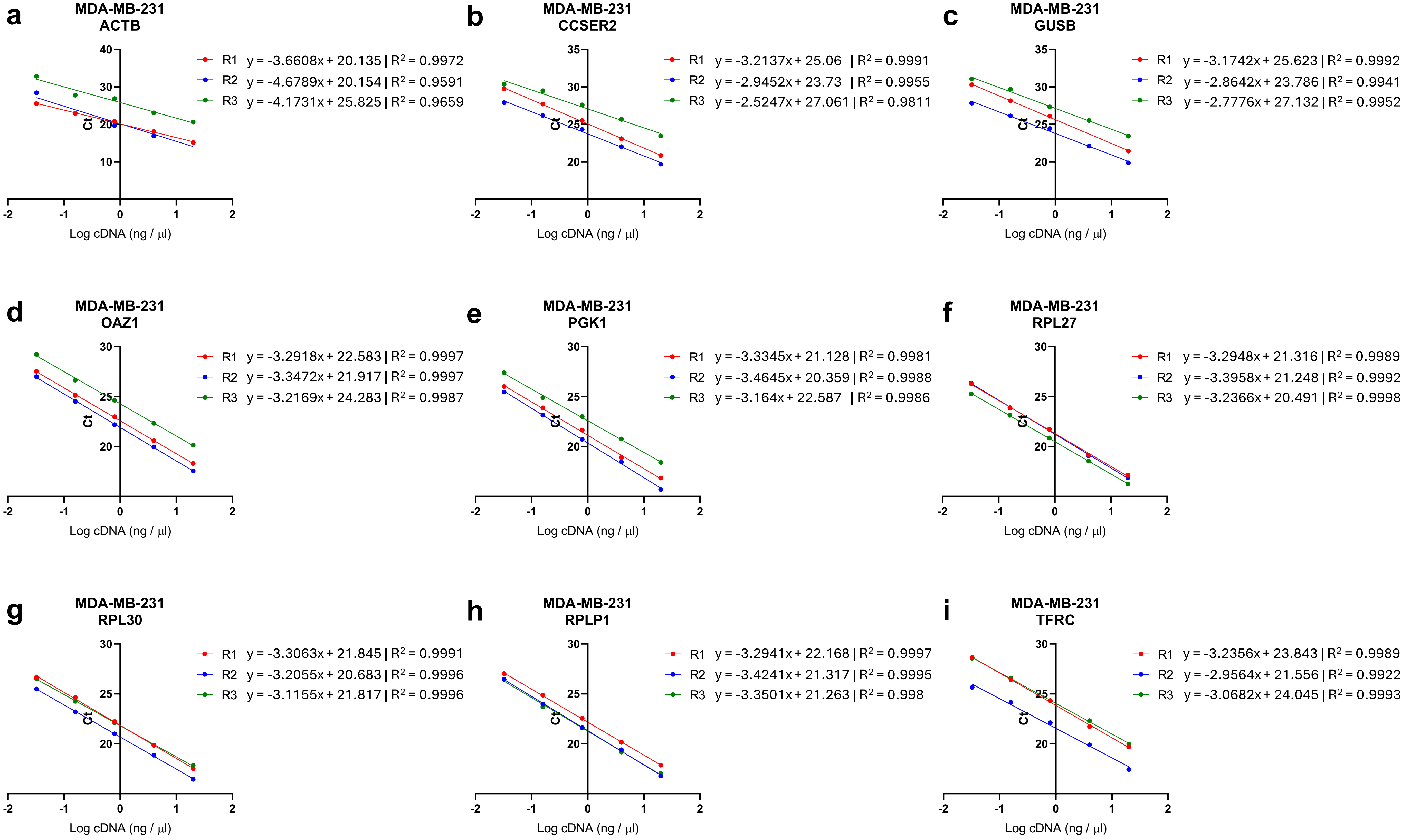

Supplementary Figure S9. Standard curves with line equations and R^2 value for (A) *ACTB,* (B) *CCSER2,* (C) *GUSB,* (D) *OAZ1,* (E) *PGK1,* (F) *RPL27,* (G) *RPL30,* (H) *RPLP1* and (I) *TFRC* in MDA-MB-231 breast cancer cells. R1, R2 and R3 refers to the biological replicate.

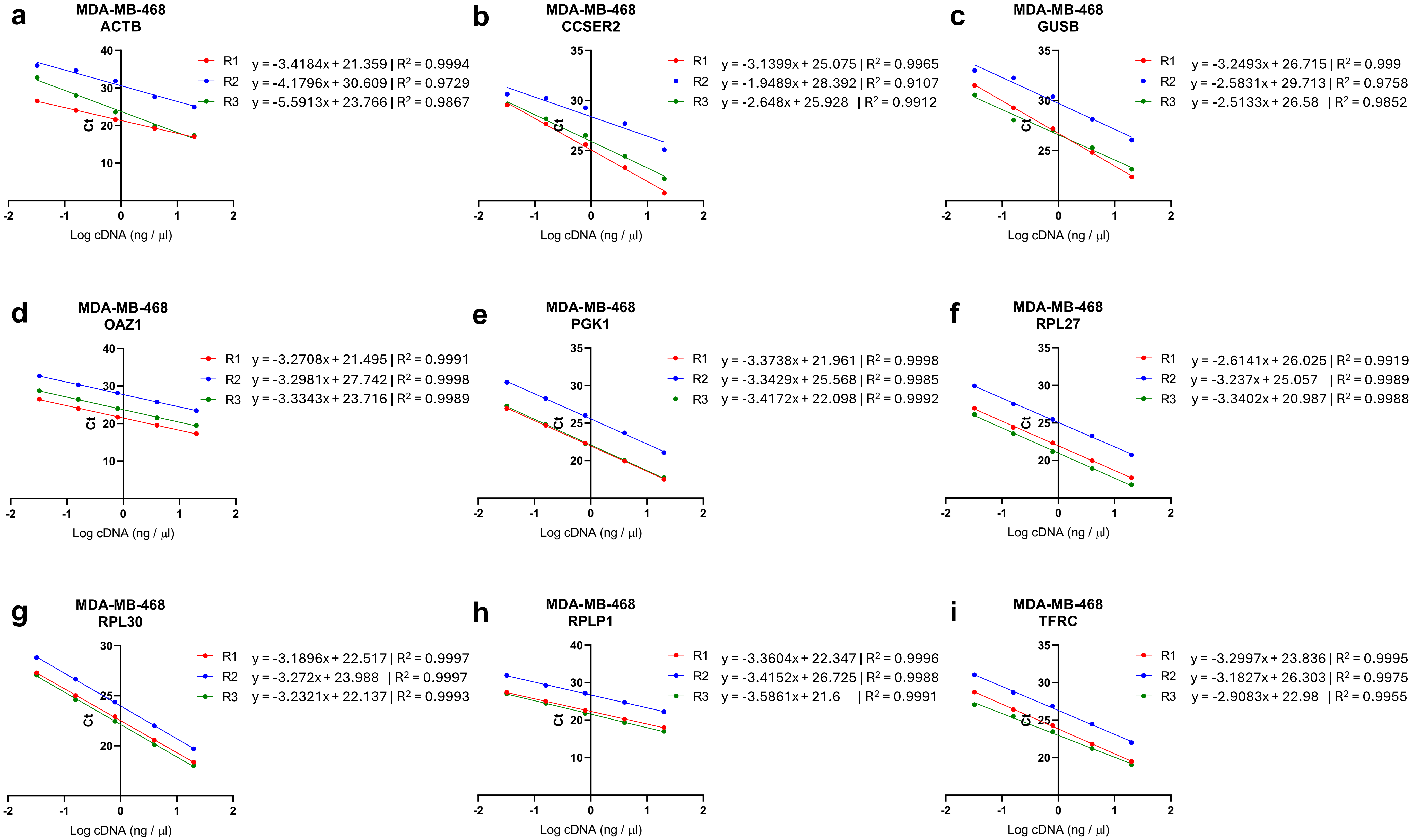

Supplementary Figure S10. Standard curves with line equations and R^2 value for (A) *ACTB,* (B) *CCSER2,* (C) *GUSB,* (D) *OAZ1,* (E) *PGK1,* (F) *RPL27,* (G) *RPL30,* (H) *RPLP1* and (I) *TFRC* in MDA-MB-468 breast cancer cells. R1, R2 and R3 refers to the biological replicate.

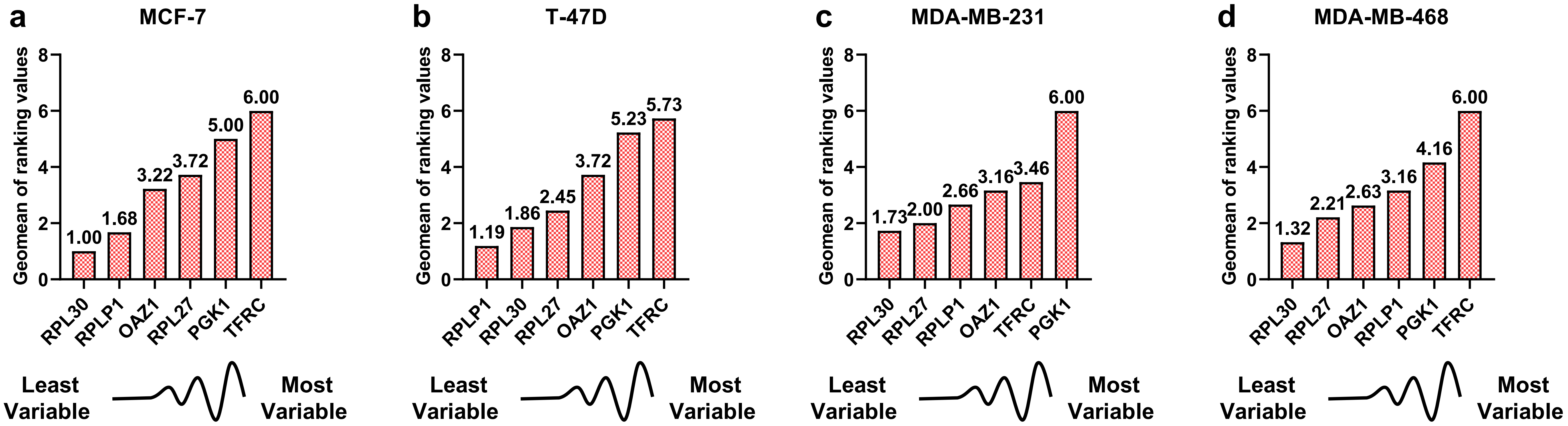

Supplementary Figure S11. Geometric mean (Geomean) of ranking values for each RG candidate according to RefFinder. The final overall ranking of RG candidates was determined by RefFinder based on the geometric mean of the weights of each gene from GeNorm, NormFinder, BestKeeper and the comparative ΔCt method for (A) MCF-7 (B) T-47D (C) MDA-MB-231 and (D) MDA-MB-468 breast cancer cell lines.
